# Prolactin Receptors and Placental Lactogen Drive Male Mouse Pancreatic Islets to Pregnancy-Related mRNA Changes

**DOI:** 10.1371/journal.pone.0121868

**Published:** 2015-03-27

**Authors:** Lotte Goyvaerts, Katleen Lemaire, Ingrid Arijs, Julien Auffret, Mikaela Granvik, Leentje Van Lommel, Nadine Binart, Peter in’t Veld, Frans Schuit, Anica Schraenen

**Affiliations:** 1 Gene Expression Unit, Department of Cellular and Molecular Medicine, KU Leuven, Leuven, Belgium; 2 Department of Clinical and Experimental Medicine, Translational Research Center for Gastrointestinal Disorders (TARGID), University Hospital Gasthuisberg, KU Leuven, Leuven, Belgium; 3 Inserm U693, Faculté de Médecine Paris-Sud, Université Paris-Sud, Le Kremlin-Bicêtre, France; 4 Department of Pathology, Vrije Universiteit Brussel, Jette, Belgium; St. Vincent's Institute, AUSTRALIA

## Abstract

Pregnancy requires a higher functional beta cell mass and this is associated with profound changes in the gene expression profile of pancreatic islets. Taking *Tph1* as a sensitive marker for pregnancy-related islet mRNA expression in female mice, we previously identified prolactin receptors and placental lactogen as key signalling molecules. Since beta cells from male mice also express prolactin receptors, the question arose whether male and female islets have the same phenotypic resilience at the mRNA level during pregnancy. We addressed this question *in vitro*, by stimulating cultured islets with placental lactogen and *in vivo*, by transplanting male or female islets into female acceptor mice. Additionally, the islet mRNA expression pattern of pregnant prolactin receptor deficient mice was compared with that of their pregnant wild-type littermates. When cultured with placental lactogen, or when transplanted in female recipients that became pregnant (day 12.5), male islets induced the ‘islet pregnancy gene signature’, which we defined as the 12 highest induced genes in non-transplanted female islets at day 12.5 of pregnancy. In addition, serotonin immunoreactivity and beta cell proliferation was also induced in these male transplanted islets at day 12.5 of pregnancy. In order to further investigate the importance of prolactin receptors in these mRNA changes we used a prolactin receptor deficient mouse model. For the 12 genes of the signature, which are highly induced in control pregnant mice, no significant induction of mRNA transcripts was found at day 9.5 of pregnancy. Together, our results support the key role of placental lactogen as a circulating factor that can trigger the pregnancy mRNA profile in both male and female beta cells.

## Introduction

Plasticity of the functional beta cell mass is a key mechanism to adjust insulin stores and rates of secretion to the needs of the organism in order to maintain normal glucose homeostasis. Adaptations of the beta cell occur at various ranges of time. First, nutrients and incretin hormones stimulate insulin secretion during a meal acutely [1]. Furthermore, chronic glucose [2–4] and incretin hormone [5, 6] stimulation determines the future secretory beta cell response and beta cell mass. In addition, long-term adaptations of beta cells occur in physiological (pregnancy) and pathophysiological conditions (obesity, acromegaly) [7, 8].

During pregnancy, the daily insulin demand of the mother increases in order to sustain fetal growth. This rising insulin demand is accommodated via structural and functional adaptations of the endocrine pancreas leading to a larger beta cell mass, enhanced insulin production and intensified insulin release [9]. To gain a better insight into the molecular mechanisms that regulate these beta cell adaptations, several laboratories performed mRNA expression profiling on islets of Langerhans isolated from non-pregnant and pregnant mice and found that the expression of a large set of genes changed during pregnancy [10–13]. For *Tph1*, encoding the rate-limiting enzyme of serotonin biosynthesis, a more than 20-fold upregulation was found and this could be mimicked in tissue culture by addition of placental lactogen (PL) [11]. PL plays a pivotal role in the islet adaptations during pregnancy and this situation can be mimicked by intra-islet secretion in RIP-mPLI transgenic mice [14]. Moreover, the onset of PL secretion occurs at the same time as the detection of the earliest adaptations of the beta cell [9, 15, 16].

Using a prolactin receptor (PRLR) heterozygous mouse model, Huang and co-workers demonstrated that PRLRs play a key role in the islet adaptations during pregnancy [17]. Moreover, this receptor mediates the actions of prolactin and PL. Multiple isoforms of this membrane-anchored receptor have been identified [18]. In mice, the PRLR is expressed as three short (PRLR-_S_1, PRLR-_S_2 and PRLR-_S_3) and one long isoform (PRLR-_L_) [19], but species differences exist for the number and length of the isoforms. For example, in rat, there is a short, an intermediate and a long isoform. In addition, sexual dimorphism is reported for PRLR expression in rat liver: livers of females have higher PRLR levels than those of males [20]. In contrast, male and female rat islets exhibited a similar expression of the long isoform of PRLR which is the predominant form expressed in rat islets [21].

In the present work we addressed the question if male islets have the intrinsic capacity to undergo the same changes in mRNA expression as compared to female islets during pregnancy. To our knowledge no information is currently available about the expression levels of the different PRLR isoforms in male mouse islets. In this study we show that male islets express mRNA of the four PRLR isoforms at the same intensity as female islets isolated from non-pregnant mice. Furthermore, we provide evidence for the induction of a largely overlapping mRNA expression change in islets of both sexes when islets are transplanted into females that are made pregnant. Furthermore, we analysed the importance of the PRLR for these changes in mRNA expression in two situations (a) *in vitro* exposure of cultured islets to PL and (b) the phenotypic changes during pregnancy of islets isolated from *Prlr* ^+/+^ and *Prlr* ^-/-^ *mice*.

## Materials and Methods

### Ethics statement

All experiments with laboratory animals were approved by the Ethical Committee Laboratory Animals (ECD) at the KU Leuven (permit numbers: p088/2008 and p124/2012) and the local ethic committee Consortium des Animaleries Paris Sud (CAPSud) (N°2012–021). The KU Leuven laboratory has the Belgian Governmental license for small animal experiments LA1210234. The animal facility in Paris was granted approval (N°C94-043-12), given by the French Administration (Ministère de l’Agriculture). Institutional guidelines for animal welfare and experimental conduct were followed.

### Animals

Islets of Langerhans were collagenase-isolated as previously described [22]. For islet transplantation female C57BL/6J mice (8–12 weeks old) were fasted overnight and intraperitoneally injected with a single dose of streptozotocin (150 mg/kg body weight) (Sigma Aldrich Chemie GmbH, Deisenhofen, Germany). When their random fed blood glucose value was above 250 mg/dl after 3 days, they were anesthetized by intraperitoneal injection of nembutal (0.01 ml/mg body wt) (Ceva, Brussels, Belgium). The left kidney was exposed through a lumbar incision and these acceptor mice were given ± 400 fresh islets of ± 12-week-old mice under the kidney capsule. The day of vaginal plug was designated as day 0.5 of pregnancy (P0.5).

Because *Prlr* ^-/-^ female mice are sterile, we treated P0.5 pregnant mice with 5 mg progesterone pellets with biodegradable carrier binder (Innovative Research of America, Toledo, OH) as this rescues the phenotype of zygote implantation [23]. It was reported before that progesterone levels in pregnant *Prlr* ^-/-^ progesterone pellet treated mice are similar to wild type pregnant *Prlr* ^+/+^ mice [23]. Because there is an increasing number of resorption sites in the *Prlr* ^-/-^ progesterone-treated mice at day 12.5 of pregnancy (P12.5) [23], we compared islet gene expression of pregnant *Prlr* ^+/+^ mice and pregnant progesterone-treated *Prlr* ^-/-^ mice at day 9.5 of pregnancy (P9.5) instead of P12.5. The mice from the *Prlr* ^-/-^ strain that were used for experiments were 13 to 20 weeks old.

### Islet monolayers

Extracellular matrix-coated (ECM) plates were produced as previously described [24]. Isolated islets were seeded in these ECM coated plates and were cultured for 7 days in RPMI medium (10% [vol./vol.] decomplemented FCS, 100 U/ml penicillin, 100 μg/ml streptomycin, 4 mmol/l glutamax) to form monolayers. On day 7, these islet monolayers were stimulated with 0 or 500 ng/ml ovine PL (oPL) (Prospec, Ness Ziona, Israel).

### RNA extraction

Total RNA from mouse islets was extracted using a kit (Absolutely RNA microprep; Stratagene, La Jolla, CA, USA) and quantity and quality were determined using a spectrophotometer (ND-1000; NanoDrop Technologies, Wilmington, DE, USA) and a bioanalyzer (2100; Agilent, Waldbronn, Germany), respectively. To obtain total RNA of gastrocnemius muscle, liver, uterus and seminal vesicle TRizol Reagent (Gibco BRL, Carlsbad, CA) was used according to the manufacturer's protocol.

### Microarray analysis

To analyse transplanted and *Prlr* ^-/-^ and *Prlr* ^+/+^ islets, total RNA (100 ng) was used to hybridise the arrays (MoGene_1.0_ST; Affymetrix) according to manufacturer’s manual 701880Rev4 as described in [22].

The Affymetrix data were analyzed in R (version 2.12.2, http://www.r-project.org/). The raw data (.CEL files) were preprocessed with robust multichip analysis [25] using the implementation in the aroma.affymetrix R package [26]. The microarray data were deposited in the Gene Expression Omnibus repository under accession numbers GSE59141 and GSE59143. For comparative analysis, linear models for microarray data [27] was performed, based on moderated t-statistics with Benjamini-Hochberg false discovery rate (FDR) correction (adjusted p-value) [28].

When comparing male and female islet grafts in non-pregnant or pregnant condition and islets of *Prlr* ^-/-^ or *Prlr* ^+/+^ mice in non-pregnant condition, all probe sets present on the MoGene_1.0_ST array were analysed. When comparing islet grafts from non-pregnant and pregnant (P12.5) mice 163 probe sets of genes, which are known to be significantly altered at P12.5 [10], were analysed. To identify genes that differ between islets of *Prlr* ^-/-^ and *Prlr* ^+/+^ mice at P9.5, 248 probe sets of genes, which are known to be significantly altered at P9.5 [10], were analysed.

### Hierarchical clustering with heat map

MultiExperiment Viewer (MEV), which is part of the TM4 Microarray Software Suite, was used to perform hierarchical clustering and to generate heat maps with the Log2 expression values or with mean centering (= value–mean of gene) for each gene [29]. The parameters used for the hierarchical clustering were the Euclidean distance and the average linkage method.

### Quantitative RT-PCR

Following cDNA synthesis using a reverse transcriptase kit (RevertAid H Minus; Fermentas, St Leon-Rot, Germany), quantitative RT-PCR (Absolute QPCR mix; Abgene-Thermo Fisher Scientific, Waltham, MA, USA) was performed on a Rotorgene (Corbett Research, Mortlake, NSW, Australia) to estimate mRNA expression of different genes. The relative mRNA expression levels were calculated with the Pfaffl method [30] and *RNA polymerase II* (*Polr2a*) was used for normalisation. For primers and probes see S1 Table. For *Tph2* we used the Taqman gene expression assay Mm00557717_m1 (Applied Biosystems, Carlsbad, CA, USA).

### Immunohistochemistry

Islet grafts were fixed in 4% (wt/vol.) paraformaldehyde and incubated with rabbit anti-serotonin (Immunostar, Hudson, WI, USA) and guinea pig anti-insulin (a gift of Dr. C. Van Schravendijk, Brussels, Belgium). Binding of primary antibodies was visualized with anti-rabbit Cy3 and anti-guinea pig fluorescein isothiocyanate (Jackson Immunoresearch, West Grove, PA, USA) and examined with a fluorescence microscope (Nikon, Brussels, Belgium) and NIS-elements imaging software (Nikon, Brussels, Belgium).

### Beta cell proliferation

Paraffin sections were incubated with monoclonal antibody Ki-67 (Mki67; Acris Antibodies, Hiddenhausen, Germany)) and guinea pig anti-insulin (a gift of Dr. Van Schravendijk, Diabetes Research Center, Vrije Universiteit Brussel, Brussels) [31]. Biotinylated anti-guinea pig and anti-rabbit Ig were used in combination with streptavidin horseradish peroxidase and alkaline phosphatase complex to detect binding. Diaminobenzidine and fuchsin-plus were used as substrates (all reagents from Dako, Glostrup, Denmark). Quantification of insulin and MKi67-insulin double positive cells was performed with a Zeiss microscope at ×400 magnification.

### Statistics

Statistical analysis was performed on experiments with n ≥ 3 animals/samples. Because the control and oPL-treated islets were isolated from the same animals, paired student’s t-tests (p < 0.05) were performed to determine, together with a fold change (FC) ≥ 2, the significant differences between these two groups. For the other quantitative RT-PCR experiments statistical significance was determined by unpaired Student’s t-test or Welch t-test depending on the variance (p < 0.05). For the microarray analysis a fold change (FC) ≥ 1.5 and p < 0.05 (FDR 5%) was used to define significance.

## Results

### Identification of a 12-gene signature for pregnancy in islets

**Fig 1:**
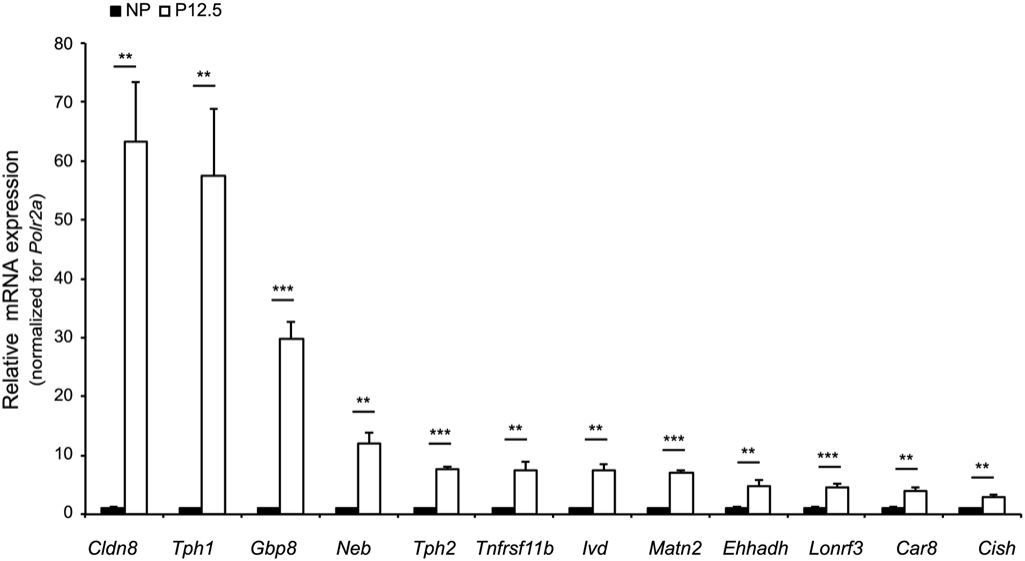
‘Islet pregnancy gene signature’ of pancreatic islets. mRNA expression of 12 genes in islets of non-pregnant female mice (NP, black bars) and at pregnancy day 12.5 (P12.5, white bars). Expression was determined via quantitative RT-PCR. The results are normalized to housekeeping gene *Polr2a* and expressed relative to the data obtained for non-pregnant islets (average = 1 for NP). Data are mean ± SEM (*n* = 5–6). *p<0.05, **p<0.01 and ***p<0.001 for difference between NP and P12.5 condition.

To identify a gene signature for pregnancy we departed from our previous work where we identified 415 differentially expressed genes during pregnancy [10]. From this gene pool, 163 genes were significantly (P < 0.001) altered at day 12.5 of pregnancy (P12.5) when compared to non-pregnant controls (S1 Fig.) [10]. For further analysis in this study, we focused on the twelve most induced protein-encoding genes at day 12.5 of pregnancy, with the omission of *Fam70a* as this was still a predicted gene at the time we started our analysis. As can be seen in Fig. 1, quantitative RT-PCR analysis confirmed pregnancy-related upregulation of the mRNA expression for all 12 selected genes. Throughout this study we will refer to this set of 12 genes as the ‘islet pregnancy gene signature’.

### Placental lactogen induces the ‘islet pregnancy gene signature’ in both male and female cultured islets

**Fig 2:**
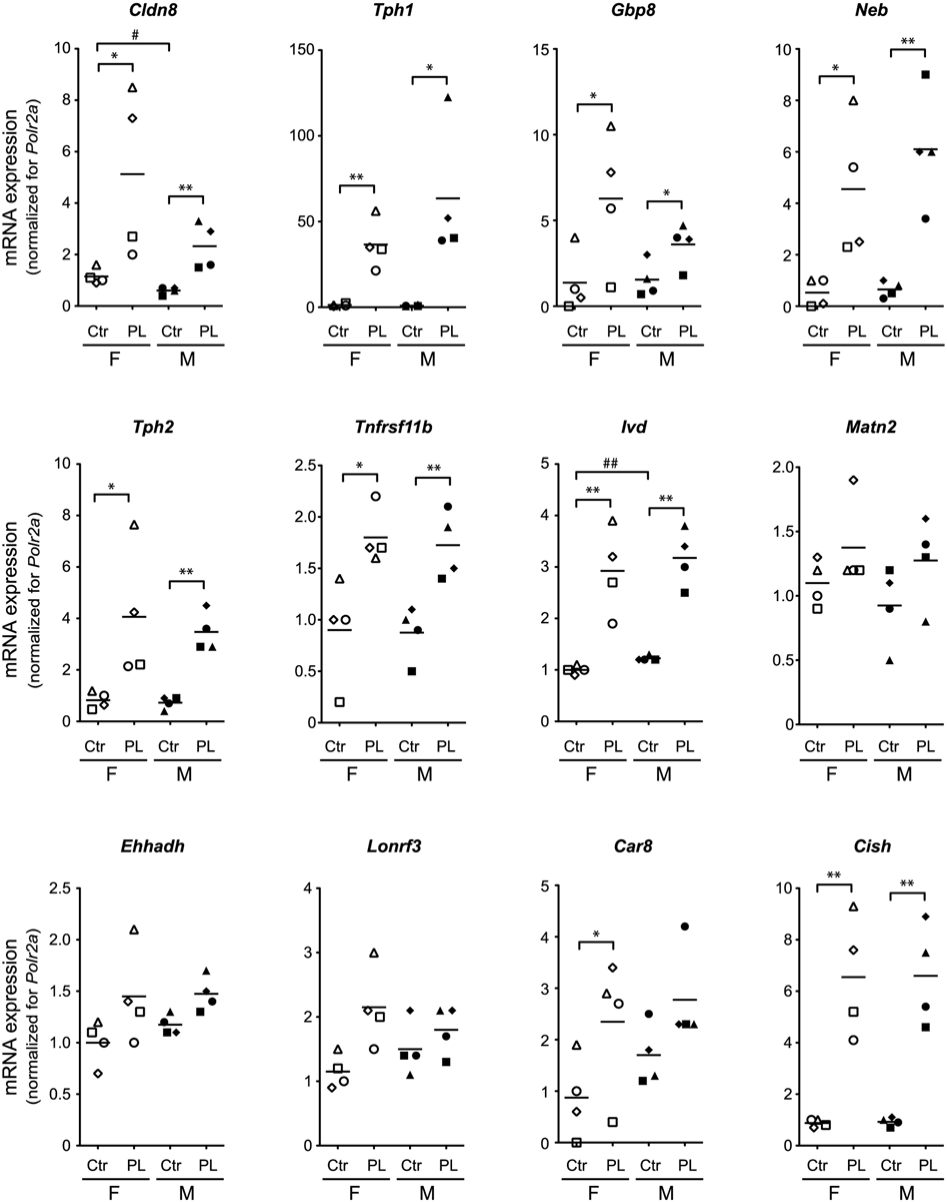
Placental lactogen induces the ‘islet pregnancy gene signature’ in cultured male and female islets. Effect of 24 h 500 ng/ml oPL on *Cldn8*, *Tph1*, *Gbp8*, *Neb*, *Tph2*, *Tnfrsf11b*, *Ivd*, *Matn2*, *Ehhadh*, *Lonrf3*, *Car8* and *Cish* in islets cultured as monolayers after isolation from non-pregnant female (F, white symbols) and male (M, black symbols) mice. The mRNA expression was determined via quantitative RT-PCR. The results are normalized to *Polr2a*, expressed relative to the data obtained for 1 sample of non-treated female islets. Each experiment (*n* = 4) is shown as a different symbol (rhombus, triangle, circle and square) and the mean is shown as a black line. Eight of the 12 tested genes could be induced (p<0.05 and FC≥2) in male and female islets with oPL treatment. *Matn2*, *Lonrf3* and *Ehhadh*, however, could not be induced by oPL in islets cultured as monolayers. For *Car8* the induction was only significant for female islets. *p<0.05, **p<0.01 and ***p<0.001 for increased expression in treated conditions compared to non-treated controls, as analysed by paired t-test and FC≥2. #p<0.05 and ##p<0.01 for difference between female and male islets (unpaired student’s t-test).

Because circulating PL plays a central role in the regulation of the islet function during pregnancy [16], we next investigated the role of PL in the induction of the ‘islet pregnancy gene signature’ in islets. Isolated islets were cultured for seven days on ECM-coated plates to form monolayers whereafter they were treated for 24 hours with 0 or 500 ng/ml oPL. Via quantitative RT-PCR, we tested all twelve genes of the ‘islet pregnancy gene signature’ in female islets isolated from non-pregnant mice and observed a significant (P<0.05 and FC ≥ 2) upregulation for 9 of the 12 genes (Fig. 2). Moreover, except for *Car8*, which did not meet the criterion of a FC ≥ 2 (Fig. 2K), the mRNA signals of the remaining eight genes were also significantly upregulated in oPL-treated islets isolated from male animals. The expression of *Matn2*, *Ehhadh* and *Lonrf3* in female as well as in male oPL-treated islets had the tendency to increase, but did not reach statistical significance (Fig. 2). These data indicate that the mRNA expression profiles of male and female islets have a similar response to oPL treatment.

### The ‘islet pregnancy gene signature’ in transplanted female and male islets

**Fig 3:**
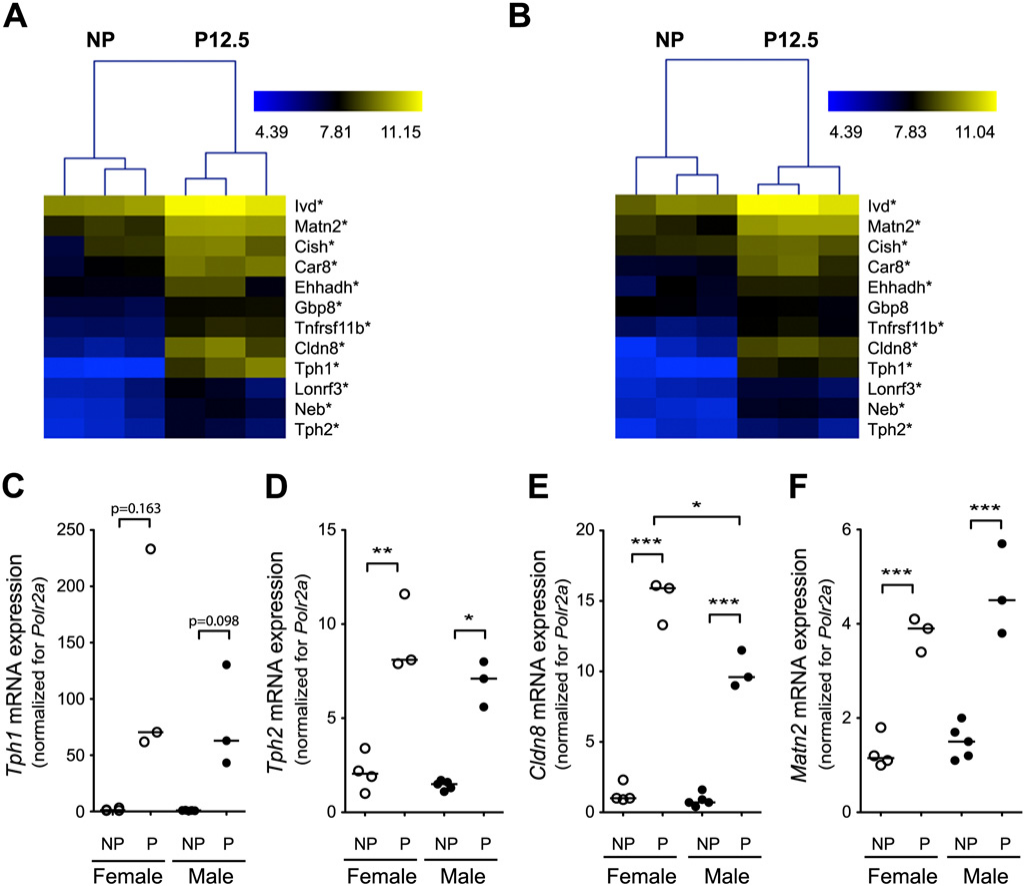
‘Islet pregnancy gene signature’ in female and male transplanted islets. Islets were isolated from female and male donors and transplanted into female recipients; at P12.5 the islet graft was removed and mRNA expression was analysed, using hierarchical clustering of microarray data and quantitative RT-PCR. A-B: heat map of the log2 values of the ‘islet pregnancy gene signature’ of female (A) and male (B) donor islets. Statistical significance (NP versus P12.5): * P<0.05 (FDR<0.05%) and FC≥1.5. *Gbp8* was not significantly induced at P12.5 in male islets (see S4 Fig.). C-F: Confirmation of pregnancy induced mRNA changes in islet grafts of male and female at P12.5 by quantitative RT-PCR for *Tph1* (C), *Tph2* (D), *Cldn8* (E) and *Matn2* (F). The results (*n* = 3–5) are normalized to housekeeping gene *Polr2a* and expressed relative to the data obtained for 1 sample of non-pregnant female transplanted islets, each sample is shown by a circle (white = female and black = male) and the mean is shown as a black line. *p<0.05, **p<0.01 and ***p<0.001 for difference between NP and P12.5 condition and between female and male.

We next studied the capacity of male islets to undergo a pregnancy-related phenotypic switch *in vivo*, using a transplantation model in which male donor islets were transferred to a streptozotocin-induced diabetic female recipient. Only recipient mice in which blood glucose levels were restored after islet transplantation were subsequently mated with a male mouse in order to obtain pregnancy. Weight and random fed blood glucose levels of the transplanted mice were similar to those of non-transplanted mice in both non-pregnant and pregnant condition (S2 Fig.). Before pregnancy, the mRNA expression profile of transplanted male and female islets was virtually identical, with significant (FC ≥ 1.5 and p < 0.05 (FDR 5%)) and expected differences being found for sex chromosome genes: Y-chromosome (*Ddx2y*, *Eif2s3y*, *Kdm5d*, *Uty*) and X-chromosome (*Xist*) (S3A Fig.). At P12.5, a robust and comparable phenotypic switch occurred both in male and female islets (Fig. 3) which is illustrated by the fact that all islet grafts of pregnant mice cluster together independent of the gender (S4 Fig.). For the ‘islet pregnancy gene signature’ a strong overlap was observed between female and male donor islets, using either microarray analysis (Fig. 3A-3B) or quantitative RT-PCR analysis (Fig. 3C-3F). A difference between male and female transplanted islets was found for *Gbp8* which was already higher in male donor islets before pregnancy (S5 Fig.). Please notice that the three genes (*Matn2*, *Lonrf3* and *Ehhadh*) that could not be induced *in vitro* by 24 hours oPL were upregulated in the *in vivo* model of female and male transplanted islets during pregnancy. When we compared the expression profile of male and female transplanted islets at P12.5 for all probe sets present on the MoGene_1.0_ST array, the only significant differences were found for the above mentioned Y chromosome genes (S3B Fig.), again indicating that male and female islets respond in the same way to pregnancy signals.

### *Prlr* mRNA expression in male islets

**Fig 4:**
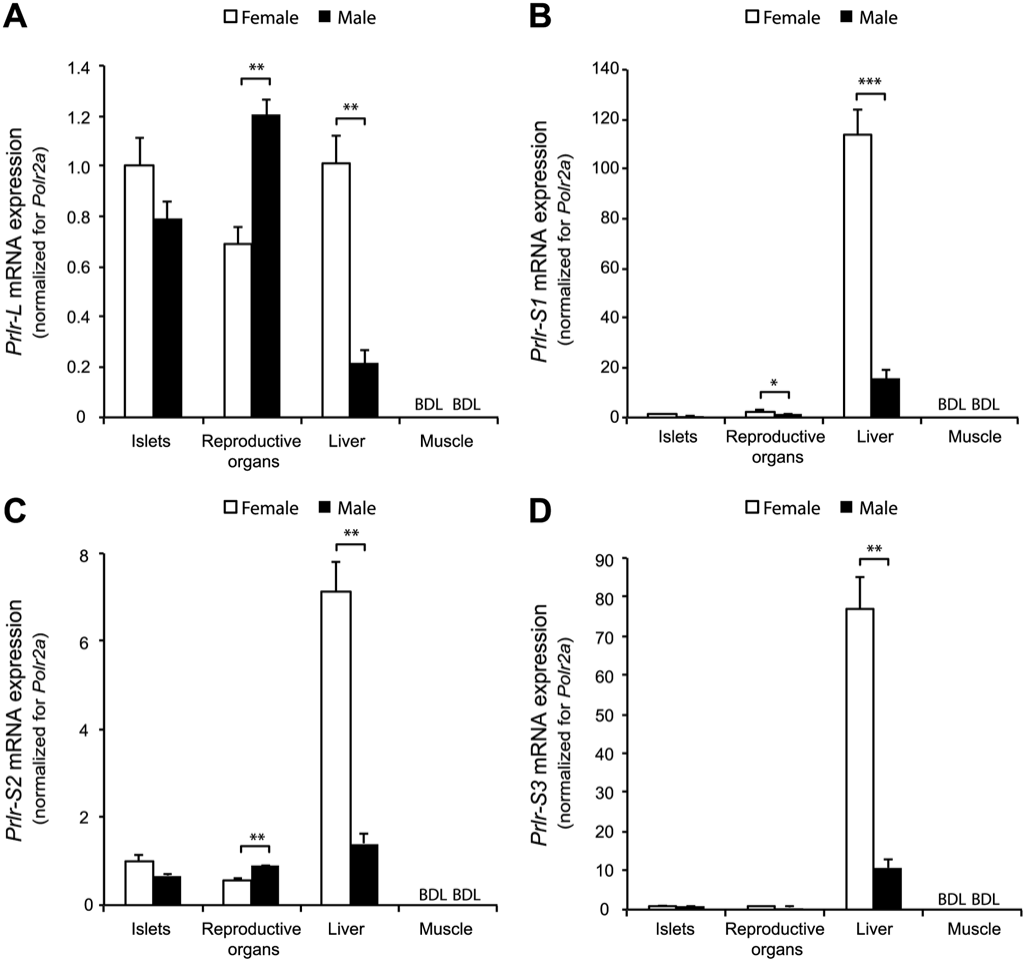
mRNA expression of the different *Prlr* transcript variants in male and female mice tissues. Quantitative RT-PCR analysis of *Prlr-*_*L*_ (A), *Prlr-*_*S1*_ (B), *Prlr-*_*S2*_ (C) and *Prlr-*_*S3*_ (D) in mouse islets, uterus, seminal vesicle, liver and muscle (gastrocnemius) isolated from females (white bars) and males (black bars). No significant difference in mRNA expression was detected between male and female islets. In liver sexual dimorphism was found for all 4 transcript variants. In muscle the signal was below the detection limit (BDL) for all 4 isoforms. The results are normalized to housekeeping gene *Polr2a* and expressed relative to the data obtained for female islets (average = 1 for female islets). Data are mean ± SEM (*n* = 4 for islets and *n* = 3 for the other tissues). *p<0.05, **p<0.01 and ***p<0.001 for difference between male and female.

A mechanism for pregnancy-related changes in male beta cells could be the expression and signal transduction of PRLR, similar to what happens in female islets during pregnancy [17]. The next step in our analysis was to investigate if male islets have the same mRNA expression profile for the four known mouse PRLR-isoforms (PRLR-_S_1, PRLR-_S_2, PRLR-_S_3 and PRLR-_L_) as female islets. In order to assess the tissue profile of the PRLR-isoforms in islets, we measured the mRNA expression of the *Prlr* transcript variants in liver, reproductive organs (seminal vesicle and uterus) and skeletal muscle of male and female mice (Fig. 4). For muscle, our negative control, the signals were for all *Prlr* transcript variants below the detection limit of the assay. Sexual dimorphism was confirmed [20] for liver, showing higher mRNA expression in female liver than in males, not only for *Prlr*-_L_ but also for *Prlr*-_S_1-3. When comparing seminal vesicles and uterus, we also observed a difference between male and female *Prlr* transcript variants. In contrast, no sexual dimorphism was found for pancreatic islets (Fig. 4) as we measured similar mRNA expression signals for the same transcript variants in both sexes.

### PRLR is required for the upregulation of the ‘islet pregnancy gene signature’

**Fig 5:**
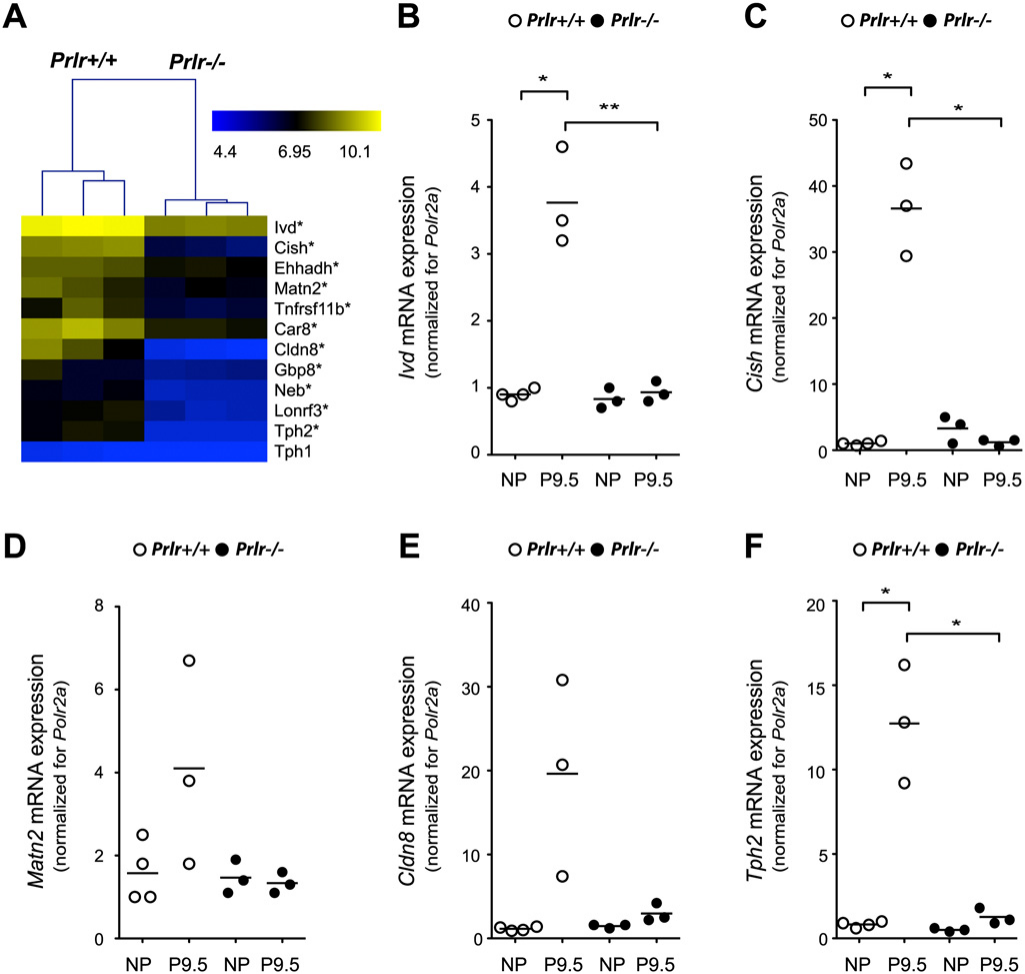
Activation of PRLR is responsible for inducing the ‘islet pregnancy gene signature’ in pancreatic islets. A: Microarray analysis was performed on RNA of islets isolated from pregnant (P9.5) *Prlr* ^+/+^ and *Prlr* ^-/-^ mice (12–21 weeks old). This figure shows the hierarchical clustering together with a heat map of the log2 values of the ‘islet pregnancy gene signature’. Statistical significance (*Prlr* ^+/+^ versus *Prlr* ^-/-^): * P<0.05 (FDR<0.05%) and FC≥1.5. B-F: quantitative RT-PCR for *Ivd* (B), *Cish* (C), *Matn2* (D), *Cldn8* (E) and *Tph2* (F) was performed on islet cDNA from *Prlr* ^+/+^ and *Prlr* ^-/-^ mice. Islets were isolated at P9.5 and from non-pregnant mice. Data (n = 3–4) are normalized to housekeeping gene *Polr2a*, expressed relative to the data obtained for 1 islet sample of a non-pregnant *Prlr* ^+/+^ mouse, each sample is shown by a circle (white = *Prlr* ^+/+^ and black = *Prlr* ^-/-^) and the mean is shown as a black line. *p<0.05, **p<0.01 and ***p<0.001 for difference between NP and P9.5 condition and for difference between *Prlr* ^+/+^ and *Prlr* ^-/-^.

To investigate if all twelve genes from the ‘islet pregnancy gene signature’ were dependent on the presence of PRLR on islets, we used a *Prlr* ^-/-^ mouse model [23] in which we compared P9.5 pregnant *Prlr* ^+/+^ mice with P9.5 pregnant progesterone-pellet treated *Prlr* ^-/-^ mice. Before analysing the mRNA expression profile during pregnancy, we investigated isolated islets from non-pregnant mice for all probe sets present on the MoGene_1.0_ST array. In this condition, the only gene with differential mRNA expression (*Prlr* ^+/+^ versus *Prlr* ^-/-^; FC ≥ 1.5, p < 0.05 and FDR 5%) was the *Prlr* gene itself (data not shown). Because the *Prlr* ^-/-^ model requires analysis at an earlier point of pregnancy (see Methods) the list of pregnancy-related mRNA changes in the islet is somewhat different as compared to P12.5 ([10] and S1 Fig.). Nevertheless, the same 12 genes from the earlier mentioned ‘islet pregnancy gene signature’ were significantly upregulated at P9.5 in wild type C57BL/6J islets. At P9.5, eleven genes of the ‘islet pregnancy gene signature’ were significantly less expressed in islets from *Prlr* ^-/-^ mice as compared to islets from *Prlr* ^+/+^ mice (Fig. 5A). In total 54 genes were found to be significantly (FC ≥ 1.5 and p < 0.05 (FDR 5%)) differentially expressed when comparing islets at P9.5 from *Prlr* ^-/-^ and *Prlr* ^+/+^ mice (S6 Fig.). For some of the genes (*Ivd*, *Cish*, *Matn2*, *Cldn8* and *Tph2*) we confirmed the microarray data via quantitative RT-PCR (Fig. 5B-5F). The difference in expression for *Matn2* and *Cldn8*, determined via quantitative RT-PCR, was not significant due to the high variance between the samples, but the trend of a decrease is present. Unexpectedly, we could not detect a difference in the *Tph1* mRNA expression when comparing P9.5 *Prlr* ^+/+^ and *Prlr* ^-/-^ islets, this because pregnant *Prlr* ^+/+^ mice did not exhibit increased *Tph1* mRNA expression (S7 Fig.).

### Male islets are serotonin competent

**Fig 6:**
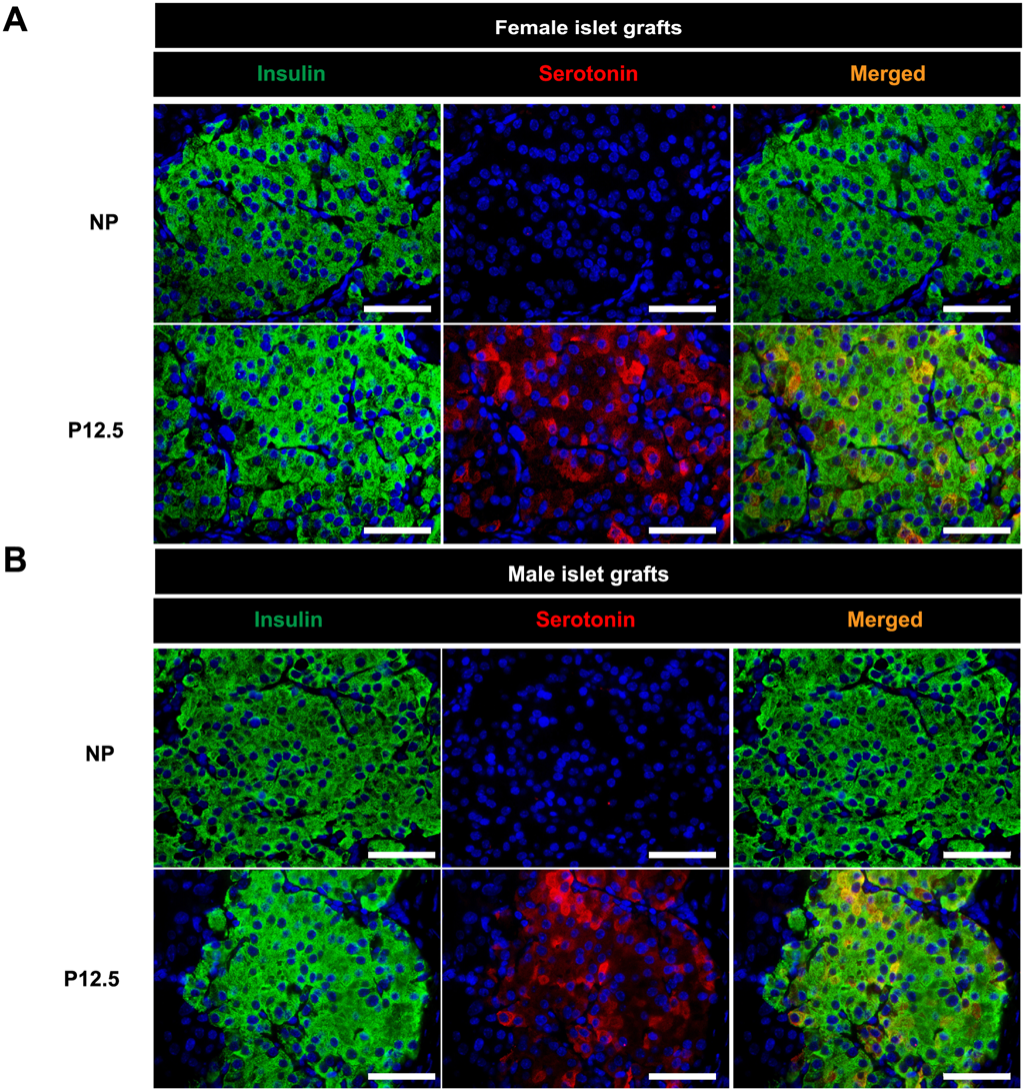
Pregnancy induces serotonin production in female and male islet grafts. Double immunostaining for insulin (green) and serotonin (red) in female (A) and male (B) islet grafts. Nuclei were stained with DAPI (blue). Presence of serotonin (red) was only detected in the samples isolated at P12.5 not in samples of the non-pregnant condition and the staining was heterogeneous. The gender of the graft did not matter as islet grafts of both genders showed serotonin staining in the pregnant condition. A magnification of 400X was used and the scale bar is 50 μm.

Since the induction of *Tph1* in islets of pregnant mice results in the synthesis of serotonin in the islets, we next performed immunohistochemistry for serotonin on male and female islet grafts from non-pregnant and pregnant mice (Fig. 6A-6B). Similar to previous observations in pancreatic islets of female non-pregnant mice [11], no serotonin could be detected in female and male islet grafts isolated from non-pregnant mice. Moreover, as we described in the pancreas of (P12.5) pregnant females [11], pregnancy induced serotonin immunoreactivity in a subpopulation of beta cells, both in female and male donor grafts. The serotonin staining was specific for the islet grafts since no serotonin signal was detected in the surrounding kidney tissue (S8 Fig.).

### Beta cell proliferation in male islet grafts during pregnancy

**Fig 7:**
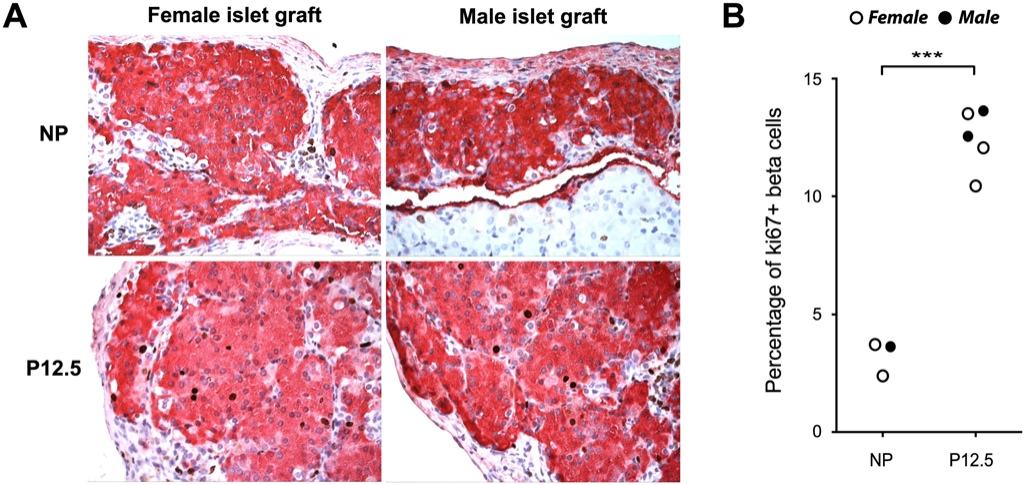
Pregnancy induces beta cell proliferation in female and male islet grafts. Double immunostaining for insulin (pink) and Ki67 (brown) in female and male islet grafts. (A) Representative images of Ki67 immunoreactivity (brown) in insulin-positive beta cells (pink) of female and male islet graft sections from non-pregnant (NP) and P12.5 mice. (B) Quantification of beta cell proliferation in islet grafts. Female islet grafts are shown as white circles and male islet grafts are shown as black circles. When gender of the donor islets was not considered, a significant difference (***p<0.001) in proliferation between NP and P12.5 condition was found.

Beta cell proliferation increases greatly in rodents during pregnancy, with a peak between the 2nd and 3rd week of gestation [9, 10, 12]. To elucidate if transplanted islets can also increase their beta cell proliferation during pregnancy, we isolated islet grafts from non-pregnant and pregnant (P12.5) mice and performed Ki67-insulin double immunostaining. Indeed, we could detect a robust and significant increase in proliferation of the grafted beta cells when the acceptor mice were pregnant (Fig. 7). Although the number of transplanted animals was insufficient to perform meaningful statistics for the male and female donor islets separately, Fig. 7B indicates that the gender of the donor material has no major influence.

## Discussion

This study supports the idea that male and female islets have a similar capacity for PRLR mediated islet phenotypic plasticity. Expression of PRLR starts in rodent islets during the perinatal period when beta cells mature [32] and high levels of PRLR remain present in pancreatic beta cells of adults [33]. In beta cells of females, where expression is known to increase during pregnancy [21], receptor stimulation by PL leads to a plethora of adaptations which together ensure an increase of the functional beta cell mass [9]. The natural ligand for this pregnancy-induced stimulation is placental lactogen, a mixture of closely homologous peptides, expressed by a cluster of tandemly repeated prolactin-like genes on chromosome 13qA3.1 of the mouse [34]. The secretion of PL by the giant trophoblast cells in the placenta parallels the changes in beta cell division and insulin secretion [9]. Studies in which islets were exposed *in vitro* to these lactogenic hormones mimic the effect of pregnancy on beta cells: increased insulin secretion, enhanced beta cell proliferation and decreased threshold of glucose stimulated insulin release [9, 11, 15, 16].

While the physiological role of PRLR on female beta cells is well documented, we know much less about the expression and potential role of PRLR in adult male islets. It was shown by Freemark and colleagues that knockdown of PRLR leads to a reduction in islet density and beta cell mass in female non-pregnant as well as in male mice [35]. Recently, it was shown that the beta cell mass of these *Prlr*-deficient mice is already impaired during embryogenesis [36]. In the current study we show that both following an *in vitro* and an *in vivo* approach PRLR stimulation induces similar mRNA expression changes in male and female islets. In the islet culture model, addition of oPL partially reproduced the ‘islet pregnancy gene signature’ we observed *in vivo*. Possible explanations for the partial *in vitro* response could be (i) the artificial extracellular matrix, (ii) the substitution of an artificial placental lactogen (oPL 500 ng/ml) for physiological mouse PL to stimulate PRLR or (iii) the lack of another circulating factor during islet culture. One example of a non-responding gene that could be matrix-related is *Matn2*, a member of the matrilins which belong to the von Willebrand factor A domain containing protein superfamily. The encoded protein is thought to be involved in the formation of filamentous networks in the extracellular matrix [37]. To compensate for the limitations of an *in vitro* model, we also studied transplanted male and female donor islets in a female recipient that was allowed to become pregnant. In this *in vivo* model, the renal graft fully reproduced the 12-gene ‘islet pregnancy gene signature’. Moreover, it seems that the gender of the donor islets does not play a role, as the male islets were also fully capable of upregulating the same set of genes that are induced in islets of female origin. Moreover, islet grafts of female as well as of male mice were able to increase their beta cell proliferation during pregnancy. This result supports the findings of previous studies in which similar functional changes were observed in male and female islets when they were exposed to lactogens ([14, 38–40]. Infusion of ovine PRL in rats lowers the threshold of glucose-stimulated insulin secretion in females as well as in males [38, 39]. Moreover the degree of insulin secretion of ovine PRL treated male rats increases to a similar extent as the one of ovine PRL treated female rats. Vasavada and colleagues state in the method section of the article describing the RIP-mPL-I mouse model that no differences were observed among age or gender [14]. These RIP-mPL-I mice have increased beta-cell proliferation, an increase in islet mass and hypoglycaemia.

For some genes of the ‘islet pregnancy gene signature’ a role in islet function was reported. Two of the 12 genes encode the rate-limiting enzyme of the serotonin biosynthesis, tryptophan hydroxylase. The induction of these two genes, *Tph1* and *Tph2*, increases the production of serotonin in islets. Our immunostaining results show that the heterogeneity that we observed for serotonin production in islets during pregnancy [11] is kept when islets are transplanted under the kidney capsule. Two recent studies investigated the role of serotonin during pregnancy in the islets via serotonin-receptor knock-out mice [13, 41]. The first study postulates that serotonin regulates beta cell proliferation via the serotonin receptor 2B [13]. This is in contrast with preliminary data of beta cell proliferation in total body *Tph1* knock-out mice [11]. The second study reported that serotonin receptor 3 influences the glucose stimulated insulin secretion of beta cells during pregnancy [41]. CISH is a member of the suppressor of cytokine signalling family and is a negative feedback regulator of the JAK2/STAT5 pathway [42]. Although this gene is upregulated during pregnancy, pancreas-specific *Cish*-deficient mice have normal glucose homeostasis and beta cell function during pregnancy [43]. *Tnfrsf11b* encodes osteoprotegerin which is reported to be a survival factor for beta cells [44]. In a recent paper however it is suggested that osteoprotegerin plays a role in promoting beta cell dysfunction [45]. The findings of our study are restricted to changes in gene expression and serotonin production to establish the role of these 12 genes in islets during pregnancy. Others and we are creating and investigating knockout mouse models to elucidate the role of this ‘pregnancy gene signature’.

Furthermore, by comparing islets isolated from pregnant *Prlr* ^+/+^ and progesterone-pellet treated *Prlr* ^-/-^ mice, we show that the ‘islet pregnancy gene signature’ is dependent on the PRLR. The *Prlr* isoform with the lowest Ct value in islets was for male and female mice *Prlr*-_L_, indicating that this isoform is the main isoform in islets. This is also the only isoform that can signal via the JAK2/STAT5 pathway. In a previous study we found putative STAT5 binding sites in the promoter of *Tph1* and *Tph2* and showed that the PL induced expression of *Tph1* is STAT5 dependent [11]. *Cish* has also been described to be a target of STAT5 and contains several consensus STAT5 binding sites (TTCNNNGAA) in its promoter [46]. In this context it was worth analysing the promoters of the other 9 genes by *in silico* search of UCSC (https://genome.ucsc.edu/). We found for all of them at least one STAT5 binding site (data not shown), suggesting that all 12 genes may be STAT5 dependent.

To our surprise, we found that the *Prlr* ^+/+^ mice, which were used for the microarray analysis, had lost the capacity to increase *Tph1* mRNA expression in islets during pregnancy. In contrast, Carol Huang showed in a recent paper that *Tph1* mRNA expression is increased during pregnancy in *Prlr* ^+/+^ mice [47]. An explanation for this difference could be the strain as the mice Carol Huang and we used were on a C57bl/6J background and a 129Sv background, respectively. Discrepancies between the 129Sv and the C57BL/6J *Prlr* strain are known: *Prlr* ^+/-^ mice on C57BL/6J pure background never lactate, not even after multiple pregnancies. In contrast, *Prlr* ^+/-^ mice on 129Sv background are capable of producing milk [48].

In the current study we concentrated on the mRNA levels of the ‘islet pregnancy gene signature’ and the role of PL for this signature. Besides lactogenic hormones, also the steroid hormones 17β-oestradiol and progesterone increase during pregnancy [49]. Our findings do not exclude a role for these other pregnancy hormones in the beta cell adaptations. It has been described that 17β-oestradiol enhances glucose-stimulated insulin secretion and insulin biosynthesis [50]. This hormone has also anti-apoptotic effects on beta cells [51]. To elucidate the role of these steroid hormones in islets during pregnancy more research is necessary. So far, genome-wide analysis of pregnancy-related changes in beta cell phenotype was focused mainly on changes in mRNA copy number per cell [10–13] and changes in non-coding RNA such as microRNAs [52]. As there are many examples of control of biological processes where the molecular mechanism is not based upon mRNA copy number but on protein abundance or post-translation modification, other approaches are needed in future studies to understand the full repertoire of pregnancy-related adaptations in mouse beta cells. The emerging field of microRNAs that regulate the translation or degradation of transcripts that are relevant for beta cell function during pregnancy [52], is interesting in this context, and further analysis of male versus female beta cells could be considered [53]. Another limitation of this work is that it was restricted to mouse beta cells. It is known from previous comparative studies between rodent and human beta cells [54, 55], that important species differences exist. Before our results can be translated into new strategies for human therapy, e.g. clinical beta cell transplantation, more information is needed about the plasticity of the functional beta cell mass during pregnancy in our own species.

In conclusion, our data support the idea that male islets, when exposed to the environmental conditions of pregnancy, undergo similar changes in gene expression as female transplanted islets. This study produced results which corroborate the finding that the key environmental factor driving the phenotypic plasticity of beta cells is a rise in circulating PL that activates PRLR on beta cells. This conclusion is supported by experiments of cultured and transplanted islets and data obtained from *Prlr* ^-/-^ mice. When extrapolated to humans, the present observation–compatibility of male and female mouse islets during pregnancy–could be of interest for the field of clinical islet transplantation and the gender of donor islets.

## Supporting Information

S1 Fig Venn diagram of pregnancy-regulated mRNA signals in female pancreatic islets. 415 genes were found to be significantly differentially expressed for at least one time-point vs. non-pregnant controls [10]. From these 415 genes, 163 and 248 genes are significantly altered, respectively, at P12.5 and P9.5, compared to NP. 124 genes that are significantly changed at P12.5 are also changed at P9.5, including the 12 genes of the ‘Islet pregnancy gene signature’ that are used throughout this article.
(TIF)

S2 Fig Body weight and random fed blood glucose levels of non-transplanted and transplanted mice. The body weight (A) of mice transplanted with female (F Tx) or male islets (M Tx) was measured in non-pregnant (NP, white bars) and pregnant condition (P12.5, black bars) and compared to the body weight of non-transplanted (non-Tx) mice. Also the random blood glucose values were monitored (B). The data are presented as mean±SEM (F Tx and M Tx, n = 3–5, non-Tx, n = 15–18).
(TIF)

S3 Fig Microarray data of four Y-chromosome and one X-chromosome gene in female and male transplanted islets. mRNA expression of *Ddx3y*, *Eif2s3y*, *Kdm5d*, *Uty* and *Xist* in non-pregnant (A) and pregnant (B) condition. Female islets are presented as white bars and male islets as black bars. Data are mean±SEM (n = 3). Statistical significance: *P<0.05 (FDR<0.05%) and FC≥1.5 and **P<0.01 (FDR<0.05%) and FC≥1.5.
(TIF)

S4 Fig mRNA expression of transplanted islets during pregnancy. The log2 values of the 163 genes that were analysed when comparing NP and P12.5 of female or male islets are presented in a heat map. Next to the data of the transplanted (Tx) islets also the log2 values of non-transplanted (non-Tx) islets from non-pregnant (NP) and pregnant (P12.5) mice are shown. The 12 genes of the ‘islet pregnancy gene signature’ are marked in red. The heat map and clustering was generated with MEV.
(PDF)

S5 Fig Microarray data of *Gbp8* mRNA expression in female and male transplanted islets. White bars represent the non-pregnant condition and black bars P12.5.
(TIF)

S6 Fig mRNA expression of *Prlr* +/+ and *Prlr-*/- islets during pregnancy. Heat map visualisation of the mRNA expression levels of the 54 genes that are significantly different between *Prlr* ^+/+^ and *Prlr* ^-/-^ islets at P9.5. The Log2 values of the microarray were normalised for each gene via mean centering using MEV. Green and red colours represent down- and upregulation respectively. The heat map and the hierarchical clustering was generated with MEV. The table next to the heat map gives the log2 values, the adjusted p-value and FC for each gene. Genes of the ‘islet pregnancy gene signature’ are marked in red.
(TIF)

S7 Fig *Tph1* mRNA expression in islets from *Prlr* ^+/+^ and *Prlr* ^-/-^ mice. A: Microarray analysis (Affymetrix MoGene_1.0_ST) of mRNA encoding *Tph1* in islets from *Prlr* ^+/+^(129Sv) (white bars), *Prlr* ^-/-^ (129Sv) (black bars) mice in non-pregnant (NP) and pregnant (P9.5) condition (mean ± SD and *n* = 3).
(TIF)

S8 Fig Immunostaining of serotonin in islet graft in non-pregnant and pregnant condition. Double immunostaining for insulin (green) and serotonin (red). Nuclei are stained with DAPI (blue). Serotonin is only detected in the insulin producing cells. Upper panel (A) female islet grafts of non-pregnant (NP) and pregnant (P12.5) mice and lower panel (B) male islet grafts of non-pregnant and pregnant (P12.5) mice. A magnification of 100X was used and the scale bar is 200 μm.
(TIF)

S1 Table Primers and probes for quantitative RT-PCR. (DOCX)

## References

[1] Henquin JC, Ravier MA, Nenquin M, Jonas JC, Gilon P. Hierarchy of the beta-cell signals controlling insulin secretion. Eur J Clin Invest. 2003;33(9):742–50. 12925032 10.1046/j.1365-2362.2003.01207.x

[2] Flamez D, Berger V, Kruhoffer M, Orntoft T, Pipeleers D, Schuit FC. Critical role for cataplerosis via citrate in glucose-regulated insulin release. Diabetes. 2002;51(7):2018–24. 12086928

[3] Bensellam M, Van Lommel L, Overbergh L, Schuit FC, Jonas JC. Cluster analysis of rat pancreatic islet gene mRNA levels after culture in low-, intermediate- and high-glucose concentrations. Diabetologia. 2009;52(3):463–76. 10.1007/s00125-008-1245-z 19165461

[4] Bonner-Weir S, Deery D, Leahy JL, Weir GC. Compensatory growth of pancreatic beta-cells in adult rats after short-term glucose infusion. Diabetes. 1989;38(1):49–53. 2642434 10.2337/diab.38.1.49

[5] Delmeire D, Flamez D, Moens K, Hinke SA, Van Schravendijk C, Pipeleers D, et al Prior in vitro exposure to GLP-1 with or without GIP can influence the subsequent beta cell responsiveness. Biochem Pharmacol. 2004;68(1):33–9.: 10.1016/j.bcp.2004.02.035 15183115

[6] Drucker DJ. Glucagon-like peptides: regulators of cell proliferation, differentiation, and apoptosis. Mol Endocrinol. 2003;17(2):161–71. 12554744 10.1210/me.2002-0306

[7] Polonsky KS. Dynamics of insulin secretion in obesity and diabetes. Int J Obes Relat Metab Disord. 2000;24 Suppl 2:S29–31. 10997604 10.1038/sj.ijo.0801273

[8] Kasayama S, Otsuki M, Takagi M, Saito H, Sumitani S, Kouhara H, et al Impaired beta-cell function in the presence of reduced insulin sensitivity determines glucose tolerance status in acromegalic patients. Clin Endocrinol (Oxf). 2000;52(5):549–55. 10792333 10.1046/j.1365-2265.2000.00986.x

[9] Parsons JA, Brelje TC, Sorenson RL. Adaptation of islets of Langerhans to pregnancy: increased islet cell proliferation and insulin secretion correlates with the onset of placental lactogen secretion. Endocrinology. 1992;130(3):1459–66. 1537300 10.1210/endo.130.3.1537300

[10] Schraenen A, de Faudeur G, Thorrez L, Lemaire K, Van Wichelen G, Granvik M, et al mRNA expression analysis of cell cycle genes in islets of pregnant mice. Diabetologia. 2010;53(12):2579–88. 10.1007/s00125-010-1912-8 20886204 PMC2974927

[11] Schraenen A, Lemaire K, de Faudeur G, Hendrickx N, Granvik M, Van Lommel L, et al Placental lactogens induce serotonin biosynthesis in a subset of mouse beta cells during pregnancy. Diabetologia. 2010;53(12):2589–99. 10.1007/s00125-010-1913-7 20938637 PMC2974930

[12] Rieck S, White P, Schug J, Fox AJ, Smirnova O, Gao N, et al The transcriptional response of the islet to pregnancy in mice. Mol Endocrinol. 2009;23(10):1702–12. 10.1210/me.2009-0144 19574445 PMC2754894

[13] Kim H, Toyofuku Y, Lynn FC, Chak E, Uchida T, Mizukami H, et al Serotonin regulates pancreatic beta cell mass during pregnancy. Nat Med. 2010;16(7):804–8. 10.1038/nm.2173 20581837 PMC2921604

[14] Vasavada RC, Garcia-Ocana A, Zawalich WS, Sorenson RL, Dann P, Syed M, et al Targeted expression of placental lactogen in the beta cells of transgenic mice results in beta cell proliferation, islet mass augmentation, and hypoglycemia. J Biol Chem. 2000;275(20):15399–406. 10809775 10.1074/jbc.275.20.15399

[15] Brelje TC, Scharp DW, Lacy PE, Ogren L, Talamantes F, Robertson M, et al Effect of homologous placental lactogens, prolactins, and growth hormones on islet B-cell division and insulin secretion in rat, mouse, and human islets: implication for placental lactogen regulation of islet function during pregnancy. Endocrinology. 1993;132(2):879–87. 8425500 10.1210/endo.132.2.8425500

[16] Sorenson RL, Brelje TC. Adaptation of islets of Langerhans to pregnancy: beta-cell growth, enhanced insulin secretion and the role of lactogenic hormones. Horm Metab Res. 1997;29(6):301–7. 10.1055/s-2007-979040 9230352

[17] Huang C, Snider F, Cross JC. Prolactin receptor is required for normal glucose homeostasis and modulation of beta-cell mass during pregnancy. Endocrinology. 2009;150(4):1618–26. 10.1210/en.2008-1003 19036882

[18] Bole-Feysot C, Goffin V, Edery M, Binart N, Kelly PA. Prolactin (PRL) and its receptor: actions, signal transduction pathways and phenotypes observed in PRL receptor knockout mice. Endocr Rev. 1998;19(3):225–68. 9626554 10.1210/edrv.19.3.0334

[19] Clarke DL, Linzer DI. Changes in prolactin receptor expression during pregnancy in the mouse ovary. Endocrinology. 1993;133(1):224–32. 8319571 10.1210/endo.133.1.8319571

[20] Kelly PA, Posner BI, Tsushima T, Friesen HG. Studies of insulin, growth hormone and prolactin binding: ontogenesis, effects of sex and pregnancy. Endocrinology. 1974;95(2):532–9. 4369224 10.1210/endo-95-2-532

[21] Moldrup A, Petersen ED, Nielsen JH. Effects of sex and pregnancy hormones on growth hormone and prolactin receptor gene expression in insulin-producing cells. Endocrinology. 1993;133(3):1165–72. 8365359 10.1210/endo.133.3.8365359

[22] Lemaire K, Ravier MA, Schraenen A, Creemers JW, Van de Plas R, Granvik M, et al Insulin crystallization depends on zinc transporter ZnT8 expression, but is not required for normal glucose homeostasis in mice. Proc Natl Acad Sci U S A. 2009;106(35):14872–7. 10.1073/pnas.0906587106 19706465 PMC2736467

[23] Binart N, Helloco C, Ormandy CJ, Barra J, Clement-Lacroix P, Baran N, et al Rescue of preimplantatory egg development and embryo implantation in prolactin receptor-deficient mice after progesterone administration. Endocrinology. 2000;141(7):2691–7. 10875275 10.1210/endo.141.7.7568

[24] Bonanno JA, Srinivas SP. Cyclic AMP activates anion channels in cultured bovine corneal endothelial cells. Exp Eye Res. 1997;64(6):953–62. 10.1006/exer.1997.0290 9301476

[25] Irizarry RA, Hobbs B, Collin F, Beazer-Barclay YD, Antonellis KJ, Scherf U, et al Exploration, normalization, and summaries of high density oligonucleotide array probe level data. Biostatistics. 2003;4(2):249–64. 10.1093/biostatistics/4.2.249 12925520

[26] Bengtsson H, Simpson K, Bullard J, Hansen K. aroma.affymetrix: A generic framework in R for analyzing small to very large Affymetrix data sets in bounded memory. Berkeley: University of California, Statistics Do; 2008 February 2008. Report No.

[27] Smyth GK. Linear models and empirical bayes methods for assessing differential expression in microarray experiments. Stat Appl Genet Mol Biol. 2004;3:Article3 10.2202/1544-6115.1027 16646809

[28] Benjamini Y, Hochberg Y. Controlling the False Discovery Rate—a Practical and Powerful Approach to Multiple Testing. J Roy Stat Soc B Met. 1995;57(1):289–300.

[29] Saeed AI, Bhagabati NK, Braisted JC, Liang W, Sharov V, Howe EA, et al TM4 microarray software suite. Methods Enzymol. 2006;411:134–93. 10.1016/S0076-6879(06)11009-5 16939790

[30] Pfaffl MW. A new mathematical model for relative quantification in real-time RT-PCR. Nucleic Acids Res. 2001;29(9):e45 11328886 10.1093/nar/29.9.e45 PMC55695

[31] In't Veld P, Lievens D, De Grijse J, Ling Z, Van der Auwera B, Pipeleers-Marichal M, et al Screening for insulitis in adult autoantibody-positive organ donors. Diabetes. 2007;56(9):2400–4. 10.2337/db07-0416 17563060

[32] Royster M, Driscoll P, Kelly PA, Freemark M. The prolactin receptor in the fetal rat: cellular localization of messenger ribonucleic acid, immunoreactive protein, and ligand-binding activity and induction of expression in late gestation. Endocrinology. 1995;136(9):3892–900. 7649097 10.1210/endo.136.9.7649097

[33] Sorenson RL, Stout LE. Prolactin receptors and JAK2 in islets of Langerhans: an immunohistochemical analysis. Endocrinology. 1995;136(9):4092–8. 7649117 10.1210/endo.136.9.7649117

[34] Wiemers DO, Shao LJ, Ain R, Dai G, Soares MJ. The mouse prolactin gene family locus. Endocrinology. 2003;144(1):313–25. 12488360 10.1210/en.2002-220724

[35] Freemark M, Avril I, Fleenor D, Driscoll P, Petro A, Opara E, et al Targeted deletion of the PRL receptor: effects on islet development, insulin production, and glucose tolerance. Endocrinology. 2002;143(4):1378–85. 11897695 10.1210/endo.143.4.8722

[36] Auffret J, Freemark MS, Carre N, Mathieu Y, Tourrel-Cuzin C, Lombes M, et al Defective prolactin signaling impairs pancreatic beta cell development during the perinatal period. Am J Physiol Endocrinol Metab. 2013 10.1152/ajpendo.00636.2012 PMC3840213 24064341

[37] Klatt AR, Becker AK, Neacsu CD, Paulsson M, Wagener R. The matrilins: modulators of extracellular matrix assembly. Int J Biochem Cell Biol. 2011;43(3):320–30. 10.1016/j.biocel.2010.12.010 21163365

[38] Sorenson RL, Johnson MG, Parsons JA, Sheridan JD. Decreased glucose stimulation threshold, enhanced insulin secretion, and increased beta cell coupling in islets of prolactin-treated rats. Pancreas. 1987;2(3):283–8. 3306662 10.1097/00006676-198705000-00006

[39] Brelje TC, Sorenson RL. Nutrient and hormonal regulation of the threshold of glucose-stimulated insulin secretion in isolated rat pancreases. Endocrinology. 1988;123(3):1582–90. 10.1210/endo-123-3-1582 3042373

[40] Cozar-Castellano I, Weinstock M, Haught M, Velazquez-Garcia S, Sipula D, Stewart AF. Evaluation of beta-cell replication in mice transgenic for hepatocyte growth factor and placental lactogen: comprehensive characterization of the G1/S regulatory proteins reveals unique involvement of p21cip. Diabetes. 2006;55(1):70–7. 16380478

[41] Ohara-Imaizumi M, Kim H, Yoshida M, Fujiwara T, Aoyagi K, Toyofuku Y, et al Serotonin regulates glucose-stimulated insulin secretion from pancreatic beta cells during pregnancy. Proc Natl Acad Sci U S A. 2013;110(48):19420–5. 10.1073/pnas.1310953110 24218571 PMC3845121

[42] Dif F, Saunier E, Demeneix B, Kelly PA, Edery M. Cytokine-inducible SH2-containing protein suppresses PRL signaling by binding the PRL receptor. Endocrinology. 2001;142(12):5286–93. 10.1210/endo.142.12.8549 11713228

[43] Jiao Y, Rieck S, Le Lay J, Kaestner KH. CISH has no non-redundant functions in glucose homeostasis or beta cell proliferation during pregnancy in mice. Diabetologia. 2013;56(11):2435–45. 10.1007/s00125-013-3014-x 23949579 PMC3816496

[44] Schrader J, Rennekamp W, Niebergall U, Schoppet M, Jahr H, Brendel MD, et al Cytokine-induced osteoprotegerin expression protects pancreatic beta cells through p38 mitogen-activated protein kinase signalling against cell death. Diabetologia. 2007;50(6):1243–7. 10.1007/s00125-007-0672-6 17443309

[45] Toffoli B, Bernardi S, Candido R, Sabato N, Carretta R, Corallini F, et al Osteoprotegerin induces morphological and functional alterations in mouse pancreatic islets. Molecular and cellular endocrinology. 2011;331(1):136–42. 10.1016/j.mce.2010.08.019 20832449

[46] Matsumoto A, Masuhara M, Mitsui K, Yokouchi M, Ohtsubo M, Misawa H, et al CIS, a cytokine inducible SH2 protein, is a target of the JAK-STAT5 pathway and modulates STAT5 activation. Blood. 1997;89(9):3148–54. 9129017

[47] Huang C. Wild-type offspring of heterozygous prolactin receptor-null female mice have maladaptive beta-cell responses during pregnancy. J Physiol. 2013;591(Pt 5):1325–38. 10.1113/jphysiol.2012.244830 23247113 PMC3607874

[48] Goffin V, Binart N, Touraine P, Kelly PA. Prolactin: the new biology of an old hormone. Annu Rev Physiol. 2002;64:47–67. 10.1146/annurev.physiol.64.081501.131049 11826263

[49] Barkley MS, Geschwind II, Bradford GE. The gestational pattern of estradiol, testosterone and progesterone secretion in selected strains of mice. Biology of reproduction. 1979;20(4):733–8. 454763 10.1095/biolreprod20.4.733

[50] Nadal A, Alonso-Magdalena P, Soriano S, Ropero AB, Quesada I. The role of oestrogens in the adaptation of islets to insulin resistance. J Physiol. 2009;587(Pt 21):5031–7. 10.1113/jphysiol.2009.177188 19687125 PMC2790246

[51] Le May C, Chu K, Hu M, Ortega CS, Simpson ER, Korach KS, et al Estrogens protect pancreatic beta-cells from apoptosis and prevent insulin-deficient diabetes mellitus in mice. Proc Natl Acad Sci U S A. 2006;103(24):9232–7. 10.1073/pnas.0602956103 16754860 PMC1482595

[52] Jacovetti C, Abderrahmani A, Parnaud G, Jonas JC, Peyot ML, Cornu M, et al MicroRNAs contribute to compensatory beta cell expansion during pregnancy and obesity. J Clin Invest. 2012;122(10):3541–51. 10.1172/JCI64151 22996663 PMC3461923

[53] Sharma S, Eghbali M. Influence of sex differences on microRNA gene regulation in disease. Biology of sex differences. 2014;5(1):3 10.1186/2042-6410-5-3 24484532 PMC3912347

[54] Eizirik DL, Pipeleers DG, Ling Z, Welsh N, Hellerstrom C, Andersson A. Major species differences between humans and rodents in the susceptibility to pancreatic beta-cell injury. Proc Natl Acad Sci U S A. 1994;91(20):9253–6. 7937750 10.1073/pnas.91.20.9253 PMC44790

[55] De Vos A, Heimberg H, Quartier E, Huypens P, Bouwens L, Pipeleers D, et al Human and rat beta cells differ in glucose transporter but not in glucokinase gene expression. J Clin Invest. 1995;96(5):2489–95. 10.1172/JCI118308 7593639 PMC185903

