# Supplementary figures and images for: Prolactin Receptors and Placental Lactogen Drive Male Mouse Pancreatic Islets to Pregnancy-Related mRNA Changes
Source: PLoS One. 2015 Mar 27;10(3):e0121868. doi: 10.1371/journal.pone.0121868 (PMC4376745; doi:10.1371/journal.pone.0121868)

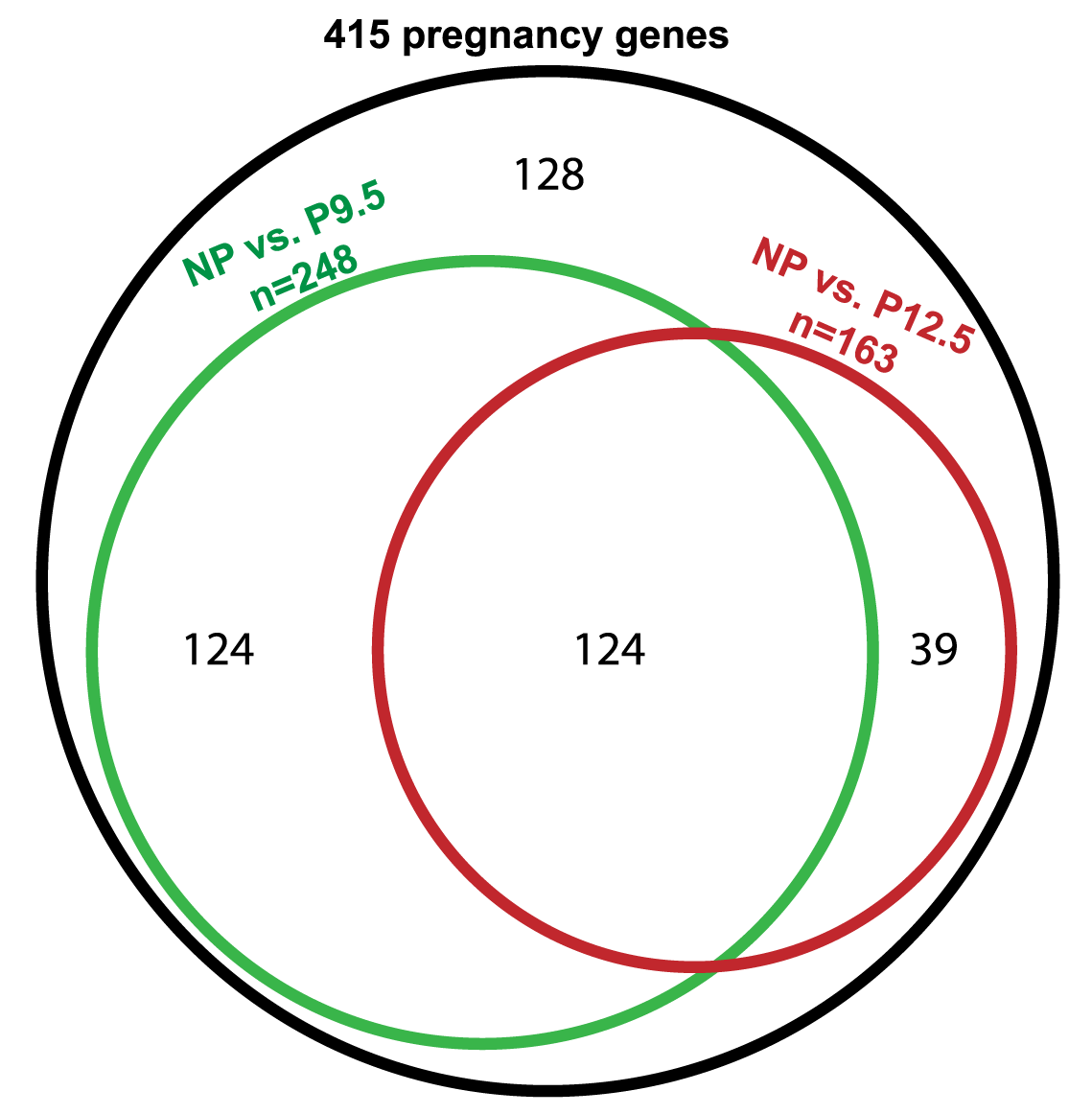

Supplement: S1 Fig — 415 genes were found to be significantly differentially expressed for at least one time-point vs. non-pregnant controls [10]. From these 415 genes, 163 and 248 genes are significantly altered, respectively, at P12.5 and P9.5, compared to NP. 124 genes that are significantly changed at P12.5 are also changed at P9.5, including the 12 genes of the ‘Islet pregnancy gene signature’ that are used throughout this article. (TIF) [file pone.0121868.s001.tif]

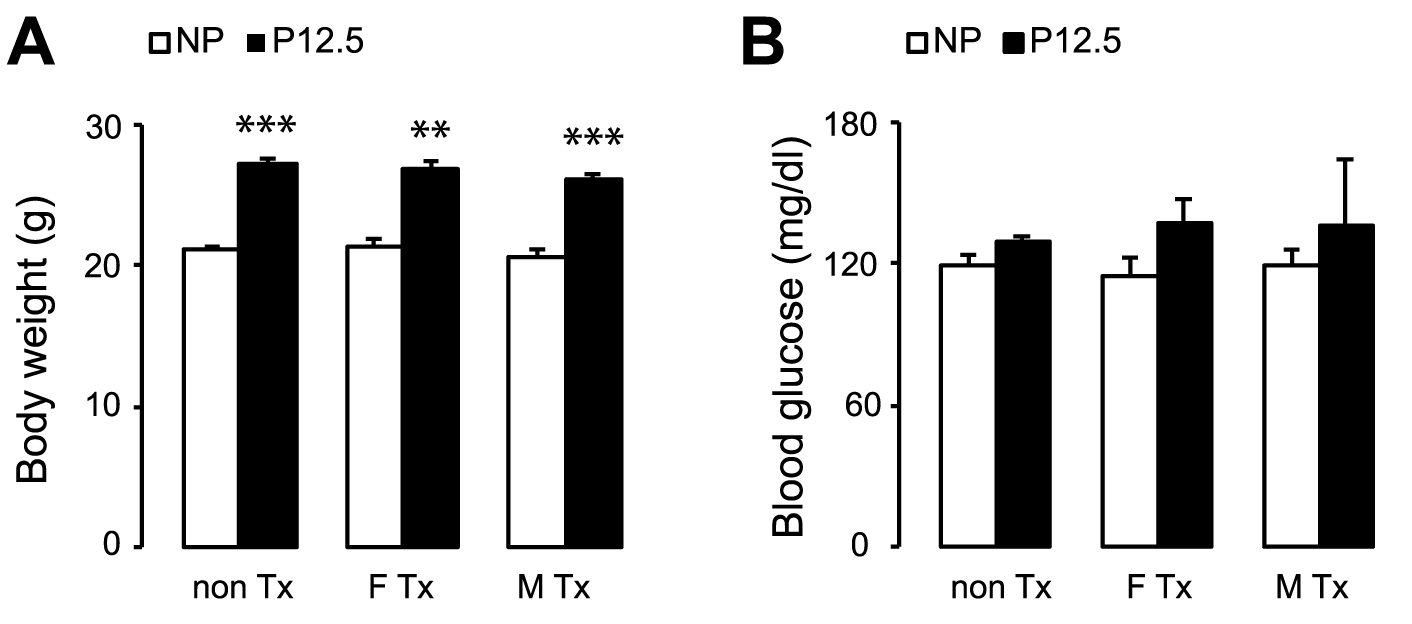

Supplement: S2 Fig — The body weight (A) of mice transplanted with female (F Tx) or male islets (M Tx) was measured in non-pregnant (NP, white bars) and pregnant condition (P12.5, black bars) and compared to the body weight of non-transplanted (non-Tx) mice. Also the random blood glucose values were monitored (B). The data are presented as mean±SEM (F Tx and M Tx, n = 3–5, non-Tx, n = 15–18). (TIF) [file pone.0121868.s002.tif]

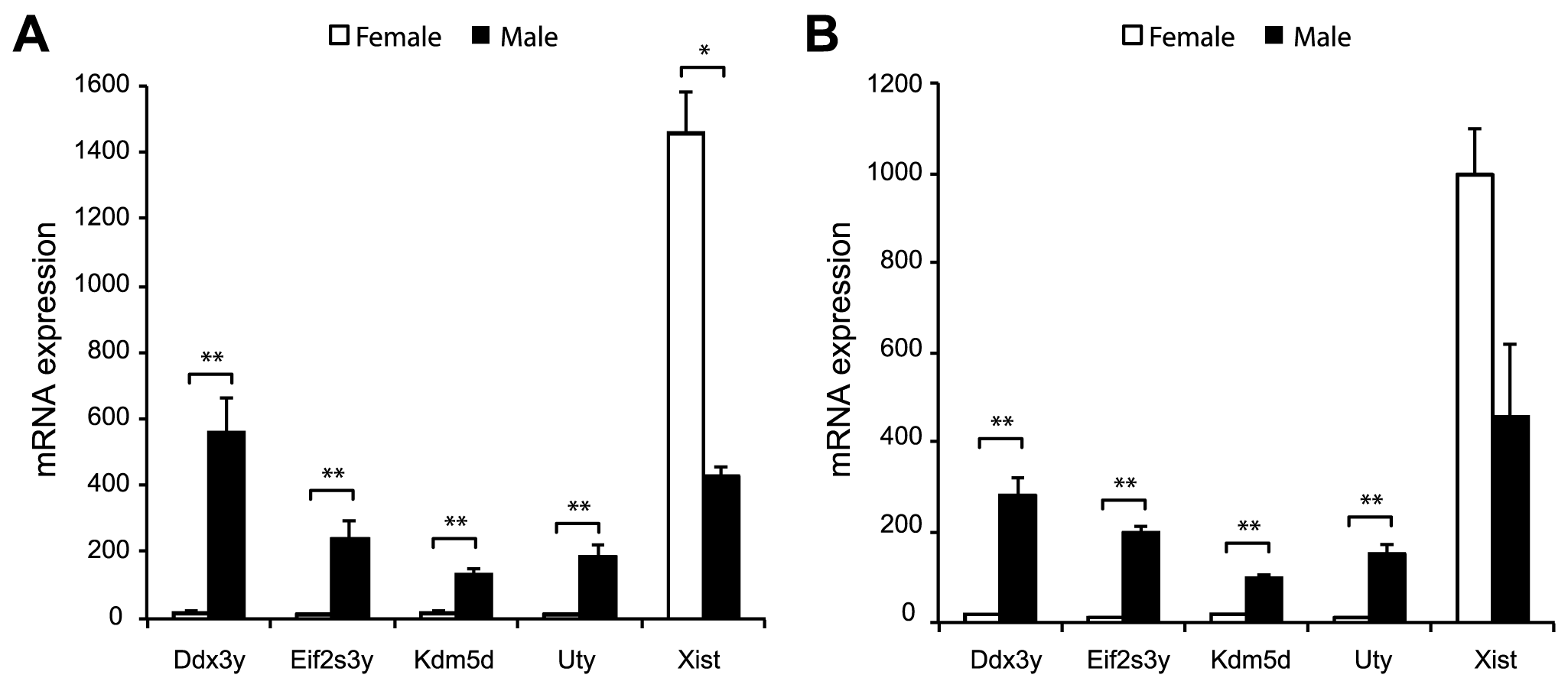

Supplement: S3 Fig — mRNA expression of Ddx3y, Eif2s3y, Kdm5d, Uty and Xist in non-pregnant (A) and pregnant (B) condition. Female islets are presented as white bars and male islets as black bars. Data are mean±SEM (n = 3). Statistical significance: *P<0.05 (FDR<0.05%) and FC≥1.5 and **P<0.01 (FDR<0.05%) and FC≥1.5. (TIF) [file pone.0121868.s003.tif]

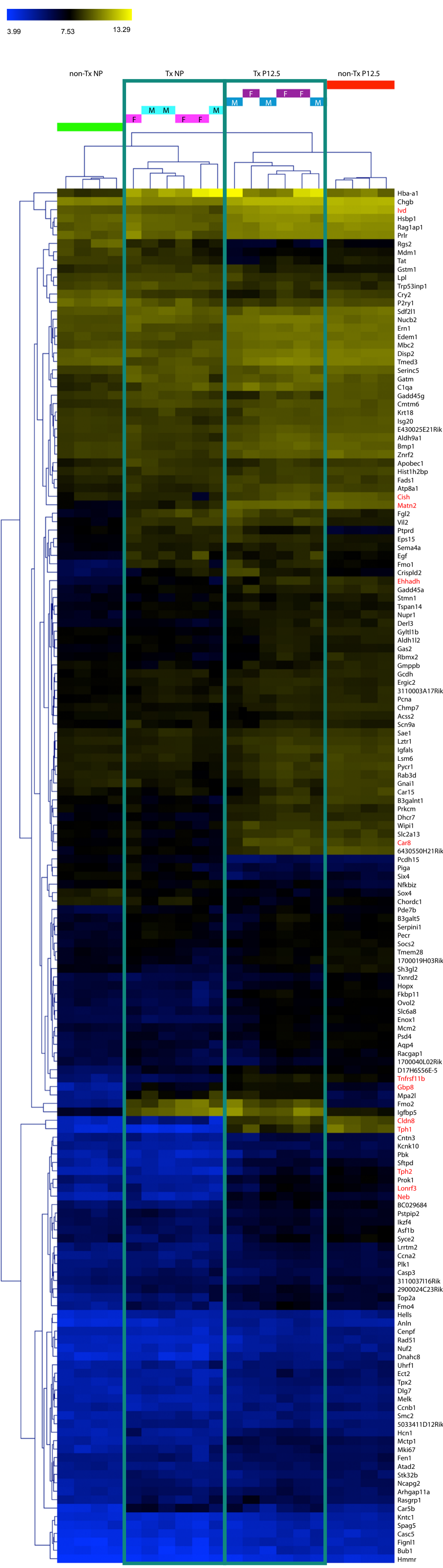

Supplement: S4 Fig — The log2 values of the 163 genes that were analysed when comparing NP and P12.5 of female or male islets are presented in a heat map. Next to the data of the transplanted (Tx) islets also the log2 values of non-transplanted (non-Tx) islets from non-pregnant (NP) and pregnant (P12.5) mice are shown. The 12 genes of the ‘islet pregnancy gene signature’ are marked in red. The heat map and clustering was generated with MEV. (PDF) [file pone.0121868.s004.pdf]

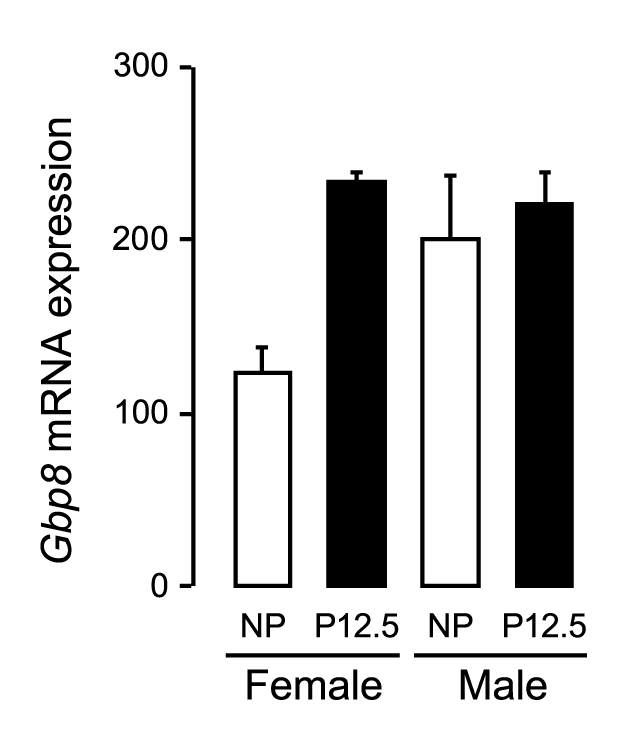

Supplement: S5 Fig — White bars represent the non-pregnant condition and black bars P12.5. (TIF) [file pone.0121868.s005.tif]

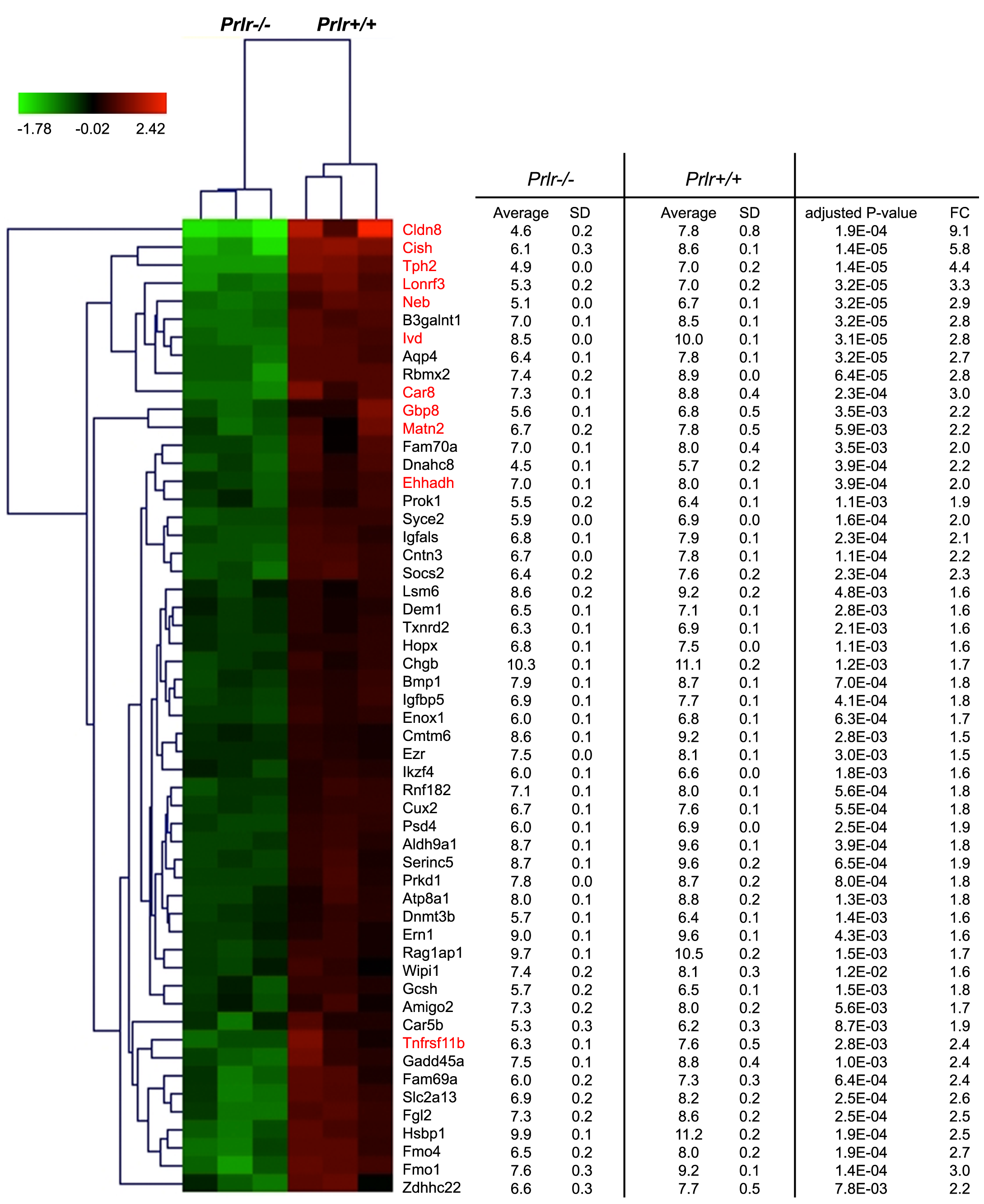

Supplement: S6 Fig — Heat map visualisation of the mRNA expression levels of the 54 genes that are significantly different between Prlr +/+ and Prlr -/- islets at P9.5. The Log2 values of the microarray were normalised for each gene via mean centering using MEV. Green and red colours represent down- and upregulation respectively. The heat map and the hierarchical clustering was generated with MEV. The table next to the heat map gives the log2 values, the adjusted p-value and FC for each gene. Genes of the ‘islet pregnancy gene signature’ are marked in red. (TIF) [file pone.0121868.s006.tif]

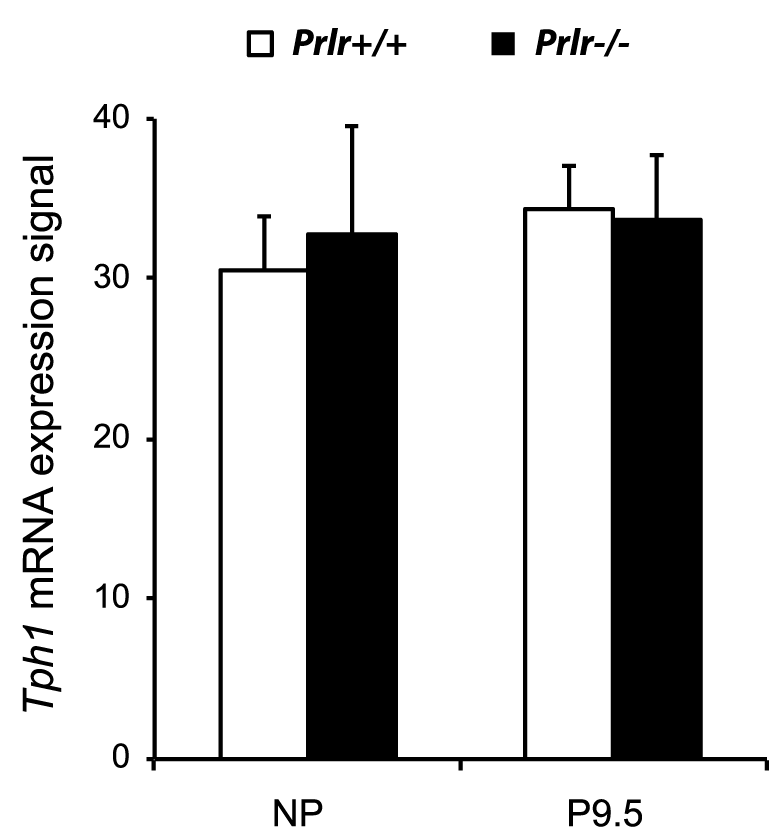

Supplement: S7 Fig — A: Microarray analysis (Affymetrix MoGene_1.0_ST) of mRNA encoding Tph1 in islets from Prlr +/+(129Sv) (white bars), Prlr -/- (129Sv) (black bars) mice in non-pregnant (NP) and pregnant (P9.5) condition (mean ± SD and n = 3). (TIF) [file pone.0121868.s007.tif]

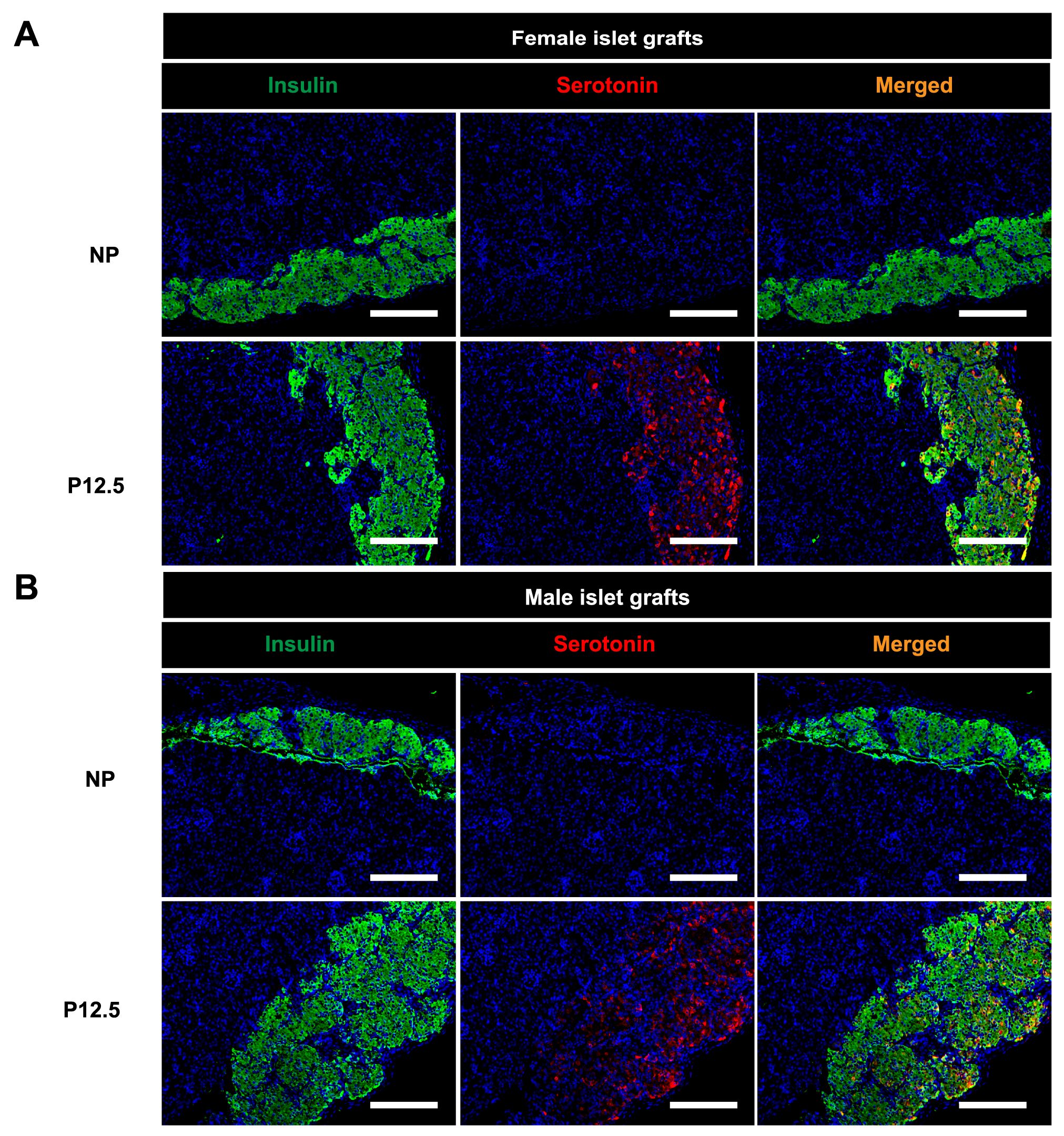

Supplement: S8 Fig — Double immunostaining for insulin (green) and serotonin (red). Nuclei are stained with DAPI (blue). Serotonin is only detected in the insulin producing cells. Upper panel (A) female islet grafts of non-pregnant (NP) and pregnant (P12.5) mice and lower panel (B) male islet grafts of non-pregnant and pregnant (P12.5) mice. A magnification of 100X was used and the scale bar is 200 μm. (TIF) [file pone.0121868.s008.tif]
