# Supplementary material for: Prolactin Receptors and Placental Lactogen Drive Male Mouse Pancreatic Islets to Pregnancy-Related mRNA Changes
Source: PLoS One. 2015 Mar 27;10(3):e0121868. doi: 10.1371/journal.pone.0121868 (PMC4376745; doi:10.1371/journal.pone.0121868)
Supplement: S1 Table — (DOCX) [file pone.0121868.s009.docx]

**Table S1: primers and probes for quantitative RT-PCR.**

| **Gene** | **Primer/probe** | **Sequence** |
| --- | --- | --- |
| *Car8* | Forward primer | 5′-AGGATATTCAATATAAGGGAA-3′ |
|  | Probe | 5′-(6-FAM)TAATCCTAACACTTTATTACCAGACCCTCT(TAMRA)-3′ |
|  | Reverse primer | 5′-CTTCATAGACCCAGTAAT-3′ |
| *Cish* | Forward primer | 5′- AAGGTGCTAGACCCTGA -3′ |
|  | Probe | 5′-(6-FAM)ATAGCCAAGACGTTCTCCTACCTTCGGGAAT(TAMRA)-3′ |
|  | Reverse primer | 5′- CTCGCTGGCTGTAATAGAA-3′ |
| *Cldn8* | Forward primer | 5′-TGGTGGATGTGGCCCTAAA-3′ |
|  | Probe | 5′-(6-FAM)GAGGGCTTCTCCCAGCTCGCG(TAMRA)-3′ |
|  | Reverse primer | 5′-CGCTGTGGTCCAGCCTATGT-3′ |
| *Ehhadh* | Forward primer | 5′-AAGCTAGTTTGGACCATACG-3′ |
|  | Probe | 5′-(6-FAM)AGCAAATGACAACTTCTGTGCAGGTGCTGA(TAMRA)-3′ |
|  | Reverse primer | 5′-CTTCTGGTATCGCTGTATTTC-3′ |
| *Gbp8* | Forward primer | 5′- AAGAACGACTTGTGGAT-3′ |
|  | Probe | 5′-(6-FAM)CATGATTCCCTGGAGAAACTACATTATGTC(TAMRA)-3′ |
|  | Reverse primer | 5′- GGATTTGGTGAAGACTTT -3′ |
| *Ivd* | Forward primer | 5′-ATGTGTTGGTAATGGAAGAGA-3′ |
|  | Probe | 5′-(6-FAM)ACTCCAACCTCTGCGTCAACCAGATTGTTC(TAMRA)-3′ |
|  | Reverse primer | 5′-CGATGAACTCACCACTGAT-3′ |
| *Lonrf3* | Forward primer | 5′- AATGCCAGAGAAGGACGAAGA-3′ |
|  | Probe | 5′-(6-FAM)ACACTGGCGGTTCTTCCTCTGGAAAGCAGA(TAMRA)-3′ |
|  | Reverse primer | 5′- AGGATACGCCGAATACCATTCAAT-3′ |
| *Matn2* | Forward primer | 5′-CAACACACCTGGCTCGTA-3′ |
|  | Probe | 5′-(6-FAM)AGCACGGATCAGAAGACTTGCAGAATCCA(TAMRA)-3′ |
|  | Reverse primer | 5′-AGACAAAGGAACCCAGCA-3′ |
| *Neb* | Forward primer | 5′-AAAGGGACAGCCATAC-3′ |
|  | Probe | 5′-(6-FAM)ATACTCCAGAACTTCGCAGAATCAAGAAAG(TAMRA)-3′ |
|  | Reverse primer | 5′- ATCCATTCGATACTTAACCT -3′ |
| *Prlr* | Forward primer | 5′- ATCATTGTGGCCGTTCTCTCT -3′ |
|  | Probe | 5′-(6-FAM) AACCCTTCAAAGCCACTGCCCAGACCAT (TAMRA)-3′ |
|  | Reverse primer  *Prlr-_L_* | 5′- GCACTCAGCAGTTCTTCAGACTTG-3’ |
|  | Reverse primer  *Prlr-_S1_* | 5′- GGGAAGTCAACTGGAGAATAGAACA-3’ |
|  | Reverse primer  *Prlr-_S2_* | 5′- TTTTCAAGTTGCTCTTTGTTGTGAA-3’ |
|  | Reverse primer  *Prlr-_S3_* | 5′- GATCCACCTTGTATTTGCTTGGAG -3′ |
| *Polr2a* | Forward primer | 5′-GCACCACGTCCAATGATATTGTG-3′ |
|  | Probe | 5′-(6-FAM)CTTCCGCACAGCCTCAATGCCCAGT(TAMRA)-3′ |
|  | Reverse primer | 5′-GGAGATGACATGGTACAGTTCTCG-3′ |
| *Tnfrsf11b* | Forward primer | 5′- CATCCAAGACATTGACCTCTGTG-3′ |
|  | Probe | 5′-(6-FAM)AGCAGCTTCGTGCCTTGATGGAGAGCCTG(TAMRA)-3′ |
|  | Reverse primer | 5′- CTTCTGGGCTGATCTTCTTCC-3′ |
| *Tph1* | Forward primer | 5′-TTCCAGGAGAATCATGTGAGC-3′ |
|  | Probe | 5′-(6-FAM)TCAACTGTTCTCGGCTGATGTCGCAGTCA(TAMRA)-3′ |
|  | Reverse primer | 5′-CATAACGTCTTCCTTCGCAGT-3′ |
